# Integration of care for hypertension and diabetes: a scoping review assessing the evidence from systematic reviews and evaluating reporting

**DOI:** 10.1186/s12913-018-3290-8

**Published:** 2018-06-20

**Authors:** Kristy C Yiu, Anke Rohwer, Taryn Young

**Affiliations:** 1 McMaster University, 1280, Hamilton, ON L8S 4L8 Canada; 2 Centre of Evidence Based Health Care, Division of Epidemiology and Biostatistics, Faculty of Medicine and Health Sciences, Stellenbosch University, Francie van Zijl Drive, Tygerberg, Cape Town, 7505 South Africa

**Keywords:** Diabetes, Hypertension, Delivery of care, Integration of care, Scoping review

## Abstract

**Background:**

With the rise in pre-mature mortality rate from non-communicable disease (NCD), there is a need for evidence-based interventions. We evaluated existing systematic reviews on effectiveness of integration of healthcare services, in particular with focus on delivery of care designed to improve health and process outcomes in people with multi-morbidity, where at least one of the conditions was diabetes or hypertension.

**Methods:**

We searched MEDLINE, EMBASE, Cochrane Library, and Health Evidence to November 8, 2016 and consulted experts. One review author screened titles, abstracts and two review authors independently screened short listed full-texts and selected reviews for inclusion. We considered systematic reviews evaluating integration of care, compared to usual care, for people with multi-morbidity. One review author extracted data and another author verified it. Two review authors independently evaluated risk of bias using ROBIS and AMSTAR. Inter-rater reliability was analysed for ROBIS and AMSTAR using Cohen’s kappa and percent agreement. The Preferred Reporting Items for Systematic Reviews and Meta-Analyses (PRISMA) checklist was used to assess reporting.

**Results:**

We identified five systematic reviews on integration of care. Four reviews focused on comorbid diabetes and depression and two covered hypertension and comorbidities of cardiovascular disease, depression, or diabetes. Interventions were poorly described. The health outcomes evaluated included risk of all-cause mortality, measures of depression, cholesterol levels, HbA1c levels, effect of depression on HbA1c levels, symptom improvement, systolic blood pressure, and hypertension control. Process outcomes included access and utilisation of healthcare services, costs, and quality of care. Overall, three reviews had a low and medium risk of bias according to ROBIS and AMSTAR respectively, while two reviews had high risk of bias as judged by both ROBIS and AMSTAR. Findings have demonstrated that collaborative care in general resulted in better health and process outcomes when compared to usual care for both depression and diabetes and hypertension and diabetes.

**Conclusions:**

Several knowledge gaps were identified on integration of care for comorbidities with diabetes and/or hypertension: limited research on this topic for hypertension, limited reviews that included primary studies based in low-middle income countries, and limited reviews on collaborative care for communicable and NCDs.

**Electronic supplementary material:**

The online version of this article (10.1186/s12913-018-3290-8) contains supplementary material, which is available to authorized users.

## Background

Globally, the number of deaths due to non-communicable diseases (NCDs) is rising [1]. Amongst NCDs, cardiovascular diseases were the leading cause of death in 2016, with 17.6 million deaths globally, while deaths due to diabetes mellitus increased by 31% from 2006 to 2016. In addition, NCDs accounted for most (80.6%) of years lived with disability (YLD) in 2016, with an increase of 17.9% from 2006 to 2016 [2]. While the leading cause of death in Sub-Saharan Africa (SSA) is still human immunodeficiency virus (HIV)/acquired immunodeficiency syndrome (AIDS), the recent release of updated results from the 2016 Global Burden of Disease study, brings focus to the increase of premature deaths caused by NCDs, such as diabetes and hypertension, and unintentional injury in SSA [1–3]. Available evidence suggests that the prevalence of diabetes has increased from less than 1% in the period of 1960–1980 to 8–13% in the 1990s [4, 5]. On the other hand, according to a 2005 study, an estimation of 10 to 20 million people out of approximately 650 million people of the general population in the SSA region were inflicted with hypertension with some communities reporting a prevalence rate as high as 38% [6–9].

In addition to the rapid rise in prevalence of both diabetes and hypertension in the SSA region, the intricate link between the two NCDs further emphasizes the importance and magnitude of this global issue. Studies have found that all persons with hypertension have an elevated risk of developing diabetes and this risk is considerably increased if the person is obese as well [10–12].

In an effort to support the African health systems in addressing this emerging problem, a series of evidence-based interventions will have to be developed and implemented to treat these co-morbidities. The Collaboration for Evidence-Based Healthcare and Public Health in Africa (CEBHA+) has developed four priority research questions in order to address this need [13]. Research on models of integrated health care delivery for hypertension and diabetes has been identified as one of the priority questions because it may provide the solution to multiple longstanding issues present in the healthcare system and the public health approach to both NCDs and communicable diseases (CDs) such as the lack of continuity of care, fragmentation of medical care/treatment process and patient education.

Despite the wide range of definitions on “integration of care” available in the literature, it could be seen that they are united by the common goals of “fostering coordination within and between healthcare organizations in order to improve patient experience, outcomes of care, and enhance overall efficiency of health systems” as proposed by Shaw et al. [14]. This was further supported by Grone and Garcia-Barbero, who defined the term as “bringing together of inputs, delivery, management and organization of services as a means [of] improving access, quality, user satisfaction and efficiency” [15]. Taking the varying definitions into account, we decided to focus on four types of integration of care: horizontal, vertical, professional (as known as integrated health services), and clinical integrations (Table 1). We only concentrated our work around these four types of integration because this study aims to examine integration at a service-delivery level for primary care, and not at an organizational or outcome level.

**Table 1:** Definitions and examples of types of integration of care

Type of Integration	Definition	Examples
Horizontal [38, 39]	Relates to strategies that link similar levels of care	Physicians join existing group practices or multiple groups merge
Vertical [38, 39]	Relates to strategies that link different levels of care	Various health care professionals, such as physicians, nurses, physiotherapists, collaborate with hospitals, universities/medical schools, health plans, etc.
Professional integration or integrated health services [38, 40]	Refers to the extent to which professionals coordinate services across various disciplines	Nurse practitioners work with dieticians to provide care for patients with diabetes
Clinical integration [38]	Refers to the extent to which care services are coordinated	Maintaining an open communication channel by having dieticians send a consultation report to the family physician after appointment with their patient

This scoping review aimed to assess the existing evidence in order to identify gaps in current knowledge on integration of care of hypertension and diabetes. Our findings will inform an appropriate research question for a systematic review which will aim to address the current knowledge gap. In addition, we compared two instruments used to assess risk of bias in systematic reviews, namely University of Bristol’s Risk of Bias of Systematic Reviews tool (ROBIS) and Assessing the Methodological Quality of Systemic Reviews tool (AMSTAR). In addition, we assessed reporting of systematic reviews according to the PRISMA guideline [16].

## Methods

The body of evidence in this scoping review was comprised solely of systematic reviews, which was defined by PRISMA as “a review of a clearly formulated question that uses systematic and explicit methods to identify, select, and critically appraise relevant research, and to collect and analyze data from the studies that are included in the review.” [16] Given that the purpose of a scoping review is to examine the existing literature on a topic and identify gaps in research, systematic reviews are an appropriate type of study for inclusion as they are considered the best form of evidence and provide a summary of existing primary studies. This evidence formed the basis of our scoping review and allowed for us to assess the scope, identify the nature, and determine the extent of systematic reviews available in current literature [17].

### Criteria for considering systematic reviews

Articles were included in this scoping review if they met the following criteria:

- Target population included people with multi-morbidities, of which diabetes and/or hypertension was one. Multi-morbidity was defined as having two or more chronic conditions for an individual.
- Interventions included integration of health care delivery, which was defined as models of care where prevention, diagnosis, or treatment of hypertension, diabetes, or any NCD was combined with the delivery of health care for any other condition (e.g. communicable disease, maternal and child care, mental health, etc.). The integration for these care services may require professional coordination across several disciplines
- The comparisons included “usual care” as defined specifically by each study and stand-alone models of health care delivery, where care was directed only towards the prevention, diagnosis, or treatment of hypertension or diabetes.
- Reported outcomes included health outcomes (e.g. all-cause mortality, disease-specific morbidity) and process outcomes (e.g. access to care, retention in care, continuity of care, quality of care, cost of care, user-views of care recipients).
- Published in English
- We included systematic reviews which had to include [18, 19]:
  - ◦ A clearly stated set of pre-determined objectives with an explicit, reproducible methodology
  - ◦ Pre-determined criteria for eligibility
  - ◦ A systematic search that attempted to identify all studies that would meet the eligibility criteria through searching through at least two data sources, with at least one of them being an electronic database
  - ◦ Performed data extraction and risk of bias assessment

### Approach for identifying systematic reviews

Medical Literature Analysis and Retrieval System Online (MEDLINE) was searched up to November 8, 2016, using medical subject heading (MeSH) terms (“diabetes mellitus”, “diabetes insipidus”, “hypertension”, “blood pressure”, “comorbidity”, “chronic disease”, “delivery of health care, integrated”, “comprehensive health care”, and other related terms). The Excerpta Medica database (EMBASE) was also searched up to November 8, 2016, using Excerpta Medica Tree (EMTREE) term (“diabetes mellitus”, “hypertension”, “diabetic hypertension”). The Cochrane Database of Systematic Reviews, its associated information management system, Archie, and the Health Evidence (http://www.healthevidence.org/) database was similarly searched for existing systematic reviews, meta-analyses, and evidence-based overviews. The search was conducted in Issue 11, November 2016 of the Cochrane Database of Systematic Reviews using the key terms “diabetes”, “hypertension”, “comorbidity”, and “delivery of health care, integrated.” Complete search strategies for all databases are provided in Additional file 1.

A search for ongoing studies was also conducted in PROSPERO, an international prospective register of systematic reviews, in February 2017. The following terms were used in the search: “collaborat*”, “integrat*”, “comorbid*”, “hypertension”, and “diabetes.” We scanned the reference lists of included systematic reviews to identify potentially relevant reviews to consider. In addition, we contacted experts in the field, thus ensuring that we did not miss any systematic review which may be of relevance.

### Selection of systematic reviews and data extraction

All citations and accompanying abstracts retrieved from the electronic searches were downloaded to an online referencing manager (RefWorks). Duplicate references were deleted before the screening process began. One reviewer (KY) screened the titles and abstracts of studies identified for potential inclusion and selected studies for inclusion using the pre-determined criteria. Full texts of potentially eligible reviews were retrieved and independently screened by two authors (KY, AR). We resolved discrepancies through discussions with the third review author (TY).

Following the study selection process, KY extracted data from the included studies and AR checked the extracted data separately. Disagreements in data abstraction were resolved through discussion and consensus. The following information was extracted from the included studies: Details of the intervention, participants, the nature of chronic disease/multi-morbidities, providers, specialist, and primary care providers, clinical setting, study designs, interventions and outcomes. The results were organized into health outcomes focused on an individual-level (e.g. all-cause mortality, disease specific morbidity) and process outcomes focused on a systems-level (e.g. access to health care, continuity of care, quality of care, cost of care, user-views of care recipients).

### Assessing risk of bias of systematic reviews

Two reviewers (AR, KY) independently assessed the risk of bias in each included review using the ROBIS and AMSTAR tools (Additional files 2 & 3). The ROBIS tool is a newly developed tool for the assessment of risk of bias for systematic reviews as opposed to those designed to assess primary studies [20]. It is composed of three phases: assessing relevance, identifying concerns with the review process, and judging risk of bias. They individually assessed each domain by describing the methods used by the study authors and determined if the study fulfilled each specific criterion within that domain by answering ‘yes’, ‘probably yes’, ‘probably no’, ‘no’, or ‘no information.’ A rating of ‘low risk of bias’, ‘high risk of bias’, or ‘unclear risk of bias’ was assigned to the overall domain after taking the fulfillment of each criterion into account. In some instances, where there was not enough evidence to support the ROBIS domains, it was assessed as unclear. Similar to the ROBIS tool, the AMSTAR tool, is used to assess the methodological quality of systematic reviews [21]. A total of 11 questions were answered with ‘yes’, ‘no’, ‘can’t answer’, or ‘not applicable’. An overall score was calculated by adding up the number of items answered with ‘yes’ which would form the numerator. The denominator was calculated by subtracting the number of ‘not applicable’ answers from 11, the total number of questions. A score of three and under was assigned a low quality. A score between four to eight was assigned a medium quality. A score of nine and above was assigned a high quality [21]. For both quality assessment tools, any discrepancies in assessment between the two reviewers were resolved through discussion and consensus. A third reviewer (TY) was consulted if an agreement could not be reached.

### Assessing reporting

The PRISMA checklist was used to check the reporting of the reviews (Additional file 4) [22]. The 27 checklist items were answered with ‘yes’, ‘partly’, ‘no’, ‘unclear’, or ‘not applicable.’ The results of the completed checklist for each of the reviews were compiled into a table. For the purposes of this study, the total number of items that was answered ‘yes’ was calculated into an overall score similar to the AMSTAR assessment.

### Data analysis

Two reviewers (AR, KY) independently collated and verified the extracted data for a descriptive synthesis of important study characteristics and results.

Inter-rater reliability for both ROBIS and AMSTAR was calculated using Statistical Package for the Social Sciences (SPSS) version 24 to determine the level of consistency between the raters’ responses in using ROBIS and AMSTAR. Cohen’s kappa was used to calculate inter-rater reliability across the domains of the tools and percent agreement was reported as a supplement to provide descriptive statistics as well for a more comprehensive result. Percent agreement was calculated by dividing the number of items in agreement by the total number of items.

The results of the completed assessments of each review using both the ROBIS and AMSTAR tools were compiled into two tables.

## Results

### Results of the search and description of included systematic reviews

**Fig. 1:**
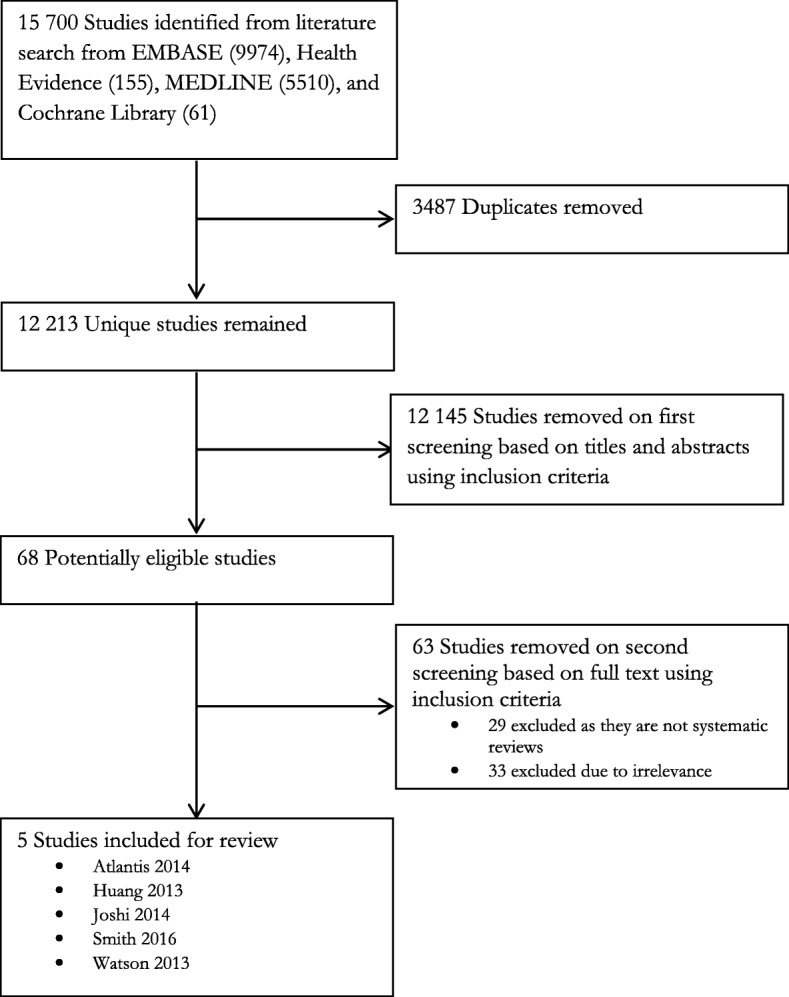
PRISMA flow chart

A total of 12,213 unique citations were identified through the literature search after duplicates were removed (Fig. 1). Of these, 12,145 citations were considered irrelevant after title/abstract screening and were directly excluded. Full texts were retrieved for 68 studies and of these, 61 were excluded with reasons (Additional file 5). One abstract without available full-text is waiting to be assessed. Twenty-eight studies were not systematic reviews and 23 did not include studies that targeted participants with comorbidities where at least one of the chronic illnesses was hypertension or diabetes.

We included five systematic reviews in this scoping review [23–27] (Fig. 1/Table 2). Three of the five systematic reviews included studies with a broad range of conditions [25–27]. Two reviews reported on hypertension and diabetes [25, 26]. One review reported on hypertension and depression [24]. Four reviews reported on diabetes and depression [23, 24, 26, 27]. For the systematic reviews that covered a broader scope and encompassed all chronic diseases in their inclusion criteria, we only extracted the information pertinent to our review.

**Table 2:** Summary of systematic review characteristics

Review	Search date	Number of studies included	Population and country where study was conducted	Intervention	Comparator	Outcomes
Atlantis 2014 [23]	August 2013	7	Adults diagnosed with depression and co-morbid diabetes Australia: 1 study USA: 6 studies RCT: 7 Range of number of participants in each study: 58–417	Integrated care: 2 studies Collaborative care: 5 studies Type of integration: Vertical Clinical	Usual care: 4 studies Enhanced usual care: 3 studies	Depression score outcome • CES-D 20 in 1 study • PHQ-9 in 2 studies • SCL-20 in 4 studies Glycaemic control by HbA1c
Huang 2013 [24]	27 March 2013	8	Patients with depression and diabetes One trial included only African Americans and two trials included only old patients (aged over 50 and 60 year old respectively) USA: all studies RCT: 8 Range of number of participants in each study: 58–417	Integrated management: 2 studies Program to Encourage Active, Rewarding Lives for Seniors (PEARLS): 1 study Multifaceted Diabetes and Depression Program (MDDP): 1 study Collaborative care: 1 study Stepped collaborative care: 1 study An individualized stepped-care depression treatment program: 1 study Improving Mood Promoting Access to Collaborative Treatment (IMPACT): 1 study Type of integration: Vertical Professional Clinical	Normal usual care: 5 trials Enhanced usual care: 3 trials	Depression treatment response and depression remission at the end of follow-up Depression treatment response at 6 and 12 months follow-up Depression remission at 6 and 12 months follow-up Diabetes clinical outcomes (HbA1c values) at the end of follow-up Diabetes clinical outcomes (HbA1c values) at 6 and 12 months follow-up Adherence to medication (including adherence of oral hypoglycemic agents and/or antidepressants)
Joshi 2014 [25]	26 May – 13 June, 2013	22 total in review; 4 of relevance	Task-shifting for the management of hypertension and cardiovascular diseases: 7 studies Task-shifting for the management of diabetes: 5 studies Cameroon: 3 studies South Africa: 1 study Before-after study: 3 RCT:1 Range of number of participants in each study: 221–1343	Task-shifting for the management of non-communicable disease Task shifting from: Physicians to health workers: 4 studies Type of integration: Vertical Professional Clinical	Usual healthcare: 4 studies	Process outcomes Disease outcomes Treatment concordance Cost-effectiveness Enablers for task-shifting Barriers to task-shifting
Smith 2016 [26]	28 Sept, 2015	18 total in review; 6 of relevance	Hypertension and depression: 1 study Depression and diabetes/heart disease: 3 studies Hypertension and diabetes: 2 studies Australia: 1 study UK: 1 study USA: 4 studies Cluster RCT: 1 RCT: 4 RCT (pilot): 1 Range of number of participants in each study: 61–400	Change to organisation of care delivery through case management or enhanced multidisciplinary team work: 5 studies Patient-oriented, such as educational or self-management support-type interventions: 3 study Type of integration: Vertical Professional Clinical	Usual care: 2 studies Usual care additional services: 3 studies Enhanced primary care: 1 studies	Patient clinical or mental health outcomes Patient-reported outcome measures Utilisation of health services Patient behaviour Provider behaviour Acceptability of the service to recipients and providers, and treatment satisfaction Economic outcomes
Watson 2013 [27]	11 June, 2012	12 studies; 3 studies of relevance	Adults (mean age = 59 years) and various chronic medical conditions, including 3 studies of relevance focusing on diabetes and depression USA: all studies RCT: 2 studies Preplanned sub-group analysis from a separate randomized controlled trial: 1 study Range of number of participants in each study: 329–417	All included studies characterized their respective intervention as a form of *collaborative care*, not another form of a practice-based intervention (such as integrated care) Type of integration: Vertical Professional Clinical	Enhanced usual care: 2 studies (9 articles) Usual care: 1 study (2 articles)	Mental health outcomes Chronic medical outcomes Harms

*RCT* randomized controlled trial, *USA* United States of America

Included systematic reviews were published between 2013 and 2016, with two published in 2013, two in 2014, and one in 2016. To better visualise the overlapping in inclusion of primary studies in the systematic reviews, a matrix table was created to present the included studies in the systematic reviews that focused on depression as a comorbidity (Additional file 6).

#### Settings

In Atlantis et al.’s review, all studies were conducted in United States of America, except one which was conducted in Australia [23]. In Huang et al.’s review, all studies were conducted in United States of America [24]. In Joshi et al.’s review, three of the relevant studies were conducted in Cameroon and one was conducted in South Africa [25]. In Smith et al.’s review, four of the relevant studies were conducted in United States of America, one in Australia, and one in United Kingdom [26]. In Watson et al.’s review, all relevant studies were conducted in United States of America [27].

#### Study design/population

Atlantis et al. included seven RCTs in its review with a participant range of 58–417. Huang et al.’s review included eight RCTs with a participant range of 58–417. Joshi et al.’s review consisted of 22 studies, of which four were relevant, including three before-after studies and one RCT with a participant range of 221–1343. Smith et al.’s review consisted of 18 studies, of which six were relevant, including one cluster RCT, four RCTs, and one pilot RCT with a participant range of 61–400. Watson et al.’s review consisted of 12 studies of which three studies were of relevance, including two RCTs and one preplanned sub-group analysis from a separate RCT with a participant range of 329–417.

#### Interventions

Brief descriptions for each review are provided in the characteristics of included systematic reviews (Table 2). While the interventions of all reviews could be broadly categorized into collaborative care, each review had a unique definition of the term. Atlantis et al. searched for ‘coordinated multidisciplinary models of care’ as the intervention. Based on Gunn et al.’s definition of collaborative care, Huang et al. defined the term as interventions that fulfilled the following four criteria: ‘a, a multi-professional patient care; b, a structured management plan; c, scheduled patient follow-up; d, enhanced inter-professional communication’ [28]. Joshi et al. focused on task-shifting, which meant a task usually performed by physicians is shifted to a different cadre of health care providers. However, since the interventions aimed to integrate health service delivery, we deemed it relevant to our scoping review. Smith et al. targeted ‘professional-, organizational-, or patient-oriented interventions’ based in primary care or community settings which aimed to improve outcomes for people with multi-morbidity. Watson et al. searched for practice-based interventions that ‘target[ed] the care process within a system of care and work[ed] to improve depression or both depression and chronic medical conditions’. All of the reviews included interventions that focused on vertical integration and clinical integration, and four on professional integration.

#### Comparison

In the majority of included reviews, the comparison was usual care or enhanced usual care, however Watson et al. also included other practice-based interventions. Usual care was defined by each study, but in general, usual care was defined by conventional/general treatments in the primary care setting. On the other hand, patients under enhanced usual care generally would receive selective parts of the intervention in additional to their usual care. For patients under the intervention arm, they received other services, such as acute treatment and relapse prevention, in addition to the services provided to those in the comparison enhanced care arm [29].

#### Outcomes

The included reviews assessed the following outcomes which could be organized into health and process outcomes. All the included reviews reported on health outcomes, while four of the five reviews reported on process outcomes. Our pre-specified health outcomes included all-cause mortality and disease specific morbidity. One review reported on all-cause mortality [27] and all of the reviews reported on disease specific morbidity. Outcomes reported under disease specific morbidity included depression outcomes [24, 26, 27], cholesterol levels [26], diabetes (HbA1c levels) clinical outcome [23, 24, 26, 27], effect of depression remission [23], symptom improvement for depression and diabetes [27], systolic blood pressure [26], and control for hypertension [25]. Our pre-specified process outcomes included access to health care, continuity of care, quality of care, cost of care, and user-views of care recipients. Two reviews reported on access and utilisation of healthcare services [25, 26]. One review reported on quality of care [27]. One review reported on cost of care [26]. None of the reviews reported on continuity of care, or user-views of care recipients. Additional outcomes that did not match our pre-specified outcomes were not included in our findings.

### Risk of bias of included systematic reviews

A summary assessment of the risk of bias of the included reviews can be found in Table 3. Overall, two of the five reviews were judged as having an overall low risk of bias after an evaluation of all domains using the ROBIS quality assessment tool. Three of the reviews were judged to have an overall high risk of bias.

**Table 3:** Summary of risk of bias for all included reviews using ROBIS

Study	Domain 1: Study eligibility criteria*	Domain 2: Identification and selection of studies^^^	Domain 3: Data collection and study appraisal^#^	Domain 4: Synthesis and findings^+^	Overall risk of bias of review	Justification
Atlantis 2014 [23]	High	Unclear	High	High	High	D1: Eligibility criteria lacked detail D2: Unclear whether authors searched for unpublished or ongoing studies and whether selection of studies was completed independently or in duplicate D3: Concerns raised about data extraction and risk of bias assessment D4: Risk of bias was not taken into consideration when conclusions were formed
Huang 2013 [24]	High	High	High	High	High	D1: No published protocol D2: Did not include sources for unpublished reports in search D3: No information on data extraction and risk of bias assessment for two of the studies included in qualitative synthesis D4: Results might have not been reported in light of risk of bias
Joshi 2014 [25]	High	High	High	High	High	D1: No protocol and lack of specific details on eligibility criteria D2: Did not consider other methods of searching for unpublished literature D3: Lack of information on quality assessment D4: Did not pre-specify methods of data analysis. Risk of bias not adequately assessed or reported with results
Smith 2016 [26]	Low	Low	Low	Low	Low	No concerns
Watson 2013 [27]	Low	Low	Low	Low	Low	The review’s eligibility criteria are limited to studies in the English language; however overall there is no concern. After deliberation, all review authors judged this review as a low risk of bias.

*Questions from Domain 1:

1.1 Did the review adhere to pre-defined objectives and eligibility criteria?

1.2 Were the eligibility criteria appropriate for the review question?

1.3 Were eligibility criteria unambiguous?

1.4 Were all restrictions in eligibility criteria based on study characteristics appropriate (e.g. date, sample size, study quality, outcomes measured)?

1.5 Were any restrictions in eligibility criteria based on sources of information appropriate (e.g.publication status or format, language, availability ofdata)?

^^^Questions from Domain 2:

2.1 Did the search include an appropriate range of databases/electronic sources for published and unpublished reports?

2.2 Were methods additional to database searching used to identify relevant reports?

2.3 Were the terms and structure of the search strategy likely to retrieve as many eligible studies as possible?

2.4 Were restrictions based on date, publication format, or language appropriate?

2.5 Were efforts made to minimise error in selection of studies?

^#^Questions from Domain 3:

3.1 Were efforts made to minimise error in data collection?

3.2 Were sufficient study characteristics available for both review authors and readers to be able to interpret the results?

3.3 Were all relevant study results collected for use in the synthesis?

3.4 Was risk of bias (or methodological quality) formally assessed using appropriate criteria?

3.5 Were efforts made to minimise error in risk of bias assessment?

^+^Questions from Domain 4:

4.1 Did the synthesis include all studies that it should?

4.2 Were all pre-defined analyses reported or departures explained?

4.3 Was the synthesis appropriate given the nature and similarity in the research questions, study designs and outcomes across included studies?

4.4 Was between-study variation (heterogeneity) minimal or addressed in the synthesis?

4.5 Were the findings robust, e.g. as demonstrated through funnel plot or sensitivity analyses?

4.6 Were biases in primary studies minimal or addressed in the synthesis?

According to the AMSTAR tool, risk of bias was judged to be medium (overall scores ranging from 5 to 8) in three reviews and high (overall scores ranging from 9 to 11) in two reviews. Table 4 presents a summary of risk of bias for all included reviews using AMSTAR.

**Table 4:** Summary of risk of bias for all included reviews using AMSTAR

AMSTAR item	Atlantis 2014 [23]	Huang 2013 [24]	Joshi 2014 [25]	Smith 2016 [26]	Watson 2013 [27]
1. ‘A priori’ design	No	No	Yes	Yes	Yes
2. Duplicate study selection and data extraction	Can’t answer	Can’t answer	No	Yes	Yes
3. Literature search	Yes	Yes	Yes	Yes	Yes
4. Status of publication	No	Can’t answer	No	Yes	Yes
5. List of studies	Yes	No	No	Yes	Yes
6. Characteristics of included studies	Yes	Yes	Yes	Yes	Yes
7. Scientific quality	Yes	Yes	No	Yes	Yes
8. Formulation of conclusion	No	No	No	Yes	Yes
9. Methods used to combine findings	Yes	Yes	Yes	Yes	Yes
10. Likelihood of publication bias	Yes	Yes	No	Yes	No
11. Conflict of interest	No	Can’t answer	No	No	Yes
Quality score	6	5	4	11	10
Quality rating	Medium	Medium	Medium	High	High

#### Comparison of risk of bias assessment using ROBIS and AMSTAR

In comparing the results of the quality assessments between ROBIS (Table 3) and AMSTAR (Table 4), all three reviews graded with an overall high risk of bias for ROBIS were judged to have a medium quality score from AMSTAR (Table 5). Meanwhile, the two reviews graded with an overall low risk of bias for ROBIS were assigned a high-quality score from AMSTAR. The extent of the agreement between the ROBIS and AMSTAR scores can also be explored by comparing the risk of bias items used in both tools (Additional file 7).

**Table 5:** Agreement between ROBIS and AMSTAR

Systematic review	ROBIS risk of bias assessment grade	AMSTAR quality assessment grade
Atlantis 2014 [23]	High risk of bias	Medium quality
Huang 2013 [24]	High risk of bias	Medium quality
Joshi 2014 [25]	High risk of bias	Medium quality
Smith 2016 [26]	Low risk of bias	High quality
Watson 2013 [27]	Low risk of bias	High quality

#### Comparison of reporting using PRISMA

The three reviews with an overall high risk of bias for ROBIS and medium AMSTAR quality scores received scores of 10, 19, and 22 out of 27 for PRISMA (Table 6) respectively. The two reviews with an overall low risk of bias for ROBIS and high AMSTAR quality scores received scores of 19 and 26 out of 27 for PRISMA respectively.

**Table 6:** Summary of completed PRISMA checklist for all included reviews

PRISMA checklist item	Atlantis 2014 [23]	Huang 2013 [24]	Joshi 2014 [25]	Smith 2016 [26]	Watson 2013 [27]
Title					
1. Title	Yes	Yes	Yes	Yes	Yes
Abstract					
2. Structured summary	Yes	Yes	Yes	Yes	Yes
Introduction					
3. Rationale	Yes	Yes	Yes	Yes	Yes
4. Objectives	Yes	Yes	Yes	Yes	Yes
Methods					
5. Protocol and registration	No	Yes	Yes	Yes	Yes
6. Eligibility criteria	Yes	Yes	Yes	Yes	Yes
7. Information sources	Yes	Yes	Yes	Yes	Yes
8. Search	Yes	Yes	No	Yes	Unclear
9. Study selection	Yes	Yes	No	Yes	Yes
10. Data collection process	Partly	Partly	No	Yes	Yes
11. Data items	No	Yes	No	Yes	Yes
12. Risk of bias in individual studies	Partly	Yes	No	Yes	Yes
13. Summary measures	Yes	Yes	N/A	Yes	Yes
14. Synthesis of results	Yes	Yes	No	Yes	Yes
15. Risk of bias across studies	Yes	Yes	No	Yes	Unclear
16. Additional analyses	Partly	Yes	No	Yes	Yes
Results					
17. Study selection	Yes	Yes	Not adequate	Yes	Yes
18. Study characteristics	Yes	Yes	Yes	Yes	Yes
19. Risk of bias within studies	Partly	Yes	No	Yes	Unclear
20. Results of individual studies	Yes	Yes	No	Yes	Yes
21. Synthesis of results	Yes	Yes	N/A	Yes	Partly
22. Risk of bias across studies	No	N/A	No	Yes	No
23. Additional analysis	Yes	N/A	N/A	N/A	No
Discussion					
24. Summary of evidence	Yes	Yes	Yes	Yes	Partly
25. Limitations	Partly	Yes	Partly	Yes	Yes
26. Conclusions	Yes	Partly	Partly	Yes	Yes
Funding					
27. Funding	Yes	No	Yes	Yes	Partly
Number of items included (yes)	19/27	22/27	10/27	26/27	19/27

#### Inter-rater reliability of ROBIS and AMSTAR

ROBIS demonstrated very poor inter-rater reliability with the majority of the questions scoring poor or no agreement. AMSTAR demonstrated better inter-rater reliability when compared to ROBIS, with majority of kappa values of questions in slight agreement or better (Additional files 8 & 9). However most of the kappa values were not statistically significant (*p* > 0.05).

### Overlap of studies included in systematic reviews

The considerable overlap in the studies included within the four systematic reviews that focused on diabetes and depression is presented in Additional file 6 where a collective total of 32 studies were included in the reviews but relate only to 19 separate studies. Ten of the studies were included in more than one review.

### Findings on the effects of integrated care

Findings on the effect of integrated care can also be found in Table 7.

**Table 7:** Findings of included studies

Comorbid conditions	Type of outcome	Findings
Diabetes and depression	Risk of all-cause mortality	6 months: RD 0.00, 95%CI -0.02 to 0.02, 2/7 studies of relevance (Watson 2013) 12 months: RD 0.00, 95%CI -0.02 to 0.01, 2/7 studies of relevance (Watson 2013)
	Depression	Depression scores SMD −0.32, 95%CI -0.53 to −0.11, 7 studies (Atlantis 2014) SMD − 0.41 95%CI -0.63 to − 0.20, 6 studies; 4 of relevance (Smith 2016) Response rate 6 months: RR 1.64, 95%CI 1.28 to 2.10, 4 studies (Huang 2013) 12 months: RR 1.42, 95%CI 1.14 to 1.76, 4 studies (Huang 2013) End of follow-up: RR 1.33, 95%CI 1.05 to 1.68, 4 studies (Huang 2013) Remission 6 months: RR 1.33, 95%CI 1.01 to 1.75, 2 studies (Huang 2013) RD 0.123, 95%CI 0.064 to 0.183, 3 studies (Watson 2013) 12 months: RR 1.20, 95%CI 0.93 to 1.55, 2 studies (Huang 2013) RD 0.077, 95%CI 0.016 to 0.137, 3 studies (Watson 2013) 18 months: RD 0.075, 95%CI 0.013 to 0.136, 3 studies (Watson 2013) 24 months: RD 0.045, 95%CI -0.023 to 0.113, 3 studies (Watson 2013) End of follow-up: RR 1.15, 95%CI 0.87 to 1.52, 2 studies (Huang 2013) Reduction (of at least 50%) in Mental Health score 6 months: RD 0.20, 95%CI 0.14 to 0.26, 4/9 studies of relevance (Watson 2013) 12 months: RD 0.17, 95%CI 0.12 to 0.23, 3/7 studies of relevance (Watson 2013) 18 months: RD 0.12, 95%CI 0.02 to 0.22, 1/3 studies of relevance (Watson 2013)
	Diabetes clinical outcome (HbA1c level)	6 months: MD − 0.06, 95%CI -0.24 to 0.12, 4 studies (Huang 2013) MD 0.13, 95%CI -0.22 to 0.48, 3 studies (Watson 2013) 12 months: MD − 0.07, 95%CI -0.28 to 0.13, 4 studies (Huang 2013) MD 0.24, 95%CI -0.14 to 0.62, 3 studies (Watson 2013) End of follow-up: WMD − 0.33, 95%CI -0.66 to 0.00%, 7 studies (Atlantis 2014) MD − 0.13, 95%CI -0.46 to 0.19, 7 studies (Huang 2013) WMD − 0.33, 95%CI -0.66 to 0.00%, 7 studies (Smith 2016)
	Effect of depression remission on HbA1c	SMD for depression scores were unable to predict the WMD in HbA1c values; p − 0.828, coefficient 0.19, 95%CI -1.93 to 2.31, 7 studies (Atlantis 2014)
	Symptom improvement	Watson et al. reported greater depression symptom improvement scores in intervention groups at 6 months: MD 0.38, 95%CI 0.24 to 0.51, 3/5 studies of relevance (Watson 2013) 12 months: MD 0.38, 95%CI 0.30 to 0.46, 3/5 studies of relevance (Watson 2013) 24 months: MD 0.18, 95%CI 0.10 to 0.26, 2/3 studies of relevance (Watson 2013)
	Systolic blood pressure	MD −3.10, 95%CI -7.26 to 1.06, 5 studies (Smith 2016)
	Access and utilisation of healthcare services	12 months: range: 42 to 84%; usual care range: 16 to 33%, 3/4 studies of relevance (Watson 2013)
	Quality of care in terms of mental health treatment satisfaction	12 months: RD 0.205, 95%CI 0.112 to 0.299, 3/4 studies of relevance (Watson 2013) 24 months: RD 0.14, 95%CI 0.06 to 0.21, 1/3 studies of relevance (Watson 2013)

### Integrated care for depression and diabetes

#### Health outcomes

##### Risk of all-cause mortality

One review reported on the risk of all-cause mortality. Watson et al. did not find any difference between collaborative and usual care for risk of all-cause mortality at 6 months (RD 0.00, 95%CI -0.02 to 0.02, 7 studies) and 12 months (RD 0.00, 95%CI -0.02 to 0.01, 7 studies). Of the seven studies included in the 6- and 12-month meta-analyses, two included participants with diabetes and depression.

#### Depression

Three reviews reported on outcomes linked to depression. Atlantis et al., Huang et al., Smith et al., and Watson et al. found that intervention groups had better results on various depression-related outcomes, such as response and remission, compared to the usual care groups.

Atlantis et al. found improved standardised depression outcomes in collaborative care group in comparison to the usual care group (SMD -0.32, 95%CI -0.53 to − 0.11, 7 studies). The sensitivity analyses considered the fixed effects model, exclusion of lower quality studies, exclusion of study outside of USA, exclusion of studies that integrated diabetes care, exclusion of studies that considered lifestyle risk factors, and exclusion of studies with less than duration of 1 year. The pooled standardised mean difference (SMD) remained similar except after the exclusion of lower quality studies (SMD -0.17, 95%CI -0.35 to 0.00, 4 studies).

Smith et al. found a decrease in depression scores (SMD -0.41 95%CI -0.63 to − 0.20, 6 studies) for participants receiving collaborative care compared to usual care. Four studies included in the meta-analysis included participants with diabetes and depression. The standardised effect sizes (SESs) for depression in PHQ-9 outcomes ranged from 0.09 to 2.24. Five of the nine outcomes suggested moderate to large effect sizes. Three of the studies included participants with diabetes and depression.

Huang et al. reported an increase in treatment response rate (RR 1.33, 95%CI 1.05 to 1.68, 4 studies) at the end of follow-up for participants in the intervention group when compared to those in the control group. In pooling the mean proportion, Huang et al. found that 44.8% of patients in the intervention group had treatment responses as compared to 34.3% of patients in the control group. At 6-month follow-up, collaborative care demonstrated significant beneficial effect (RR 1.64, 95%CI 1.28 to 2.10, 4 studies). And at 12-month follow-up, collaborative care also demonstrated significant beneficial effect (RR 1.42, 95%CI 1.14 to 1.76, 4 studies).

Huang et al. also found that collaborative care had a non-significant effect on the rate of depression remission (RR 1.15, 95%CI 0.87 to 1.52, 2 studies). At 6-month follow-up, collaborative care demonstrated a significant increase in depression remission (RR 1.33, 95%CI 1.01 to 1.75, 2 studies). However, it should be noted that although both studies found an increased treatment response in the intervention group, neither was significant. At 12-month follow-up, it was shown that collaborative care had a non-significant effect on depression remission (RR 1.20, 95%CI 0.93 to 1.55, 2 studies).

Watson et al. also reported remission of depression in favour of collaborative care at 6 months (RD 0.123, 95%CI 0.064 to 0.183, 3 studies), 12 months (RD 0.077, 95%CI 0.016 to 0.137, 3 studies), 18 months (RD 0.075, 95%CI 0.013 to 0.136, 3 studies), and 24 months (RD 0.045, 95%CI -0.023 to 0.113, 3 studies). Of the studies included in each of the 6, 12, 18, and 24 months meta-analyses, one included participants with diabetes and depression.

Watson et al. reported reduction (of at least 50%) in Mental Health score in favour of collaborative care at 6 months (RD 0.20, 95%CI 0.14 to 0.26, 9 studies), 12 months (RD 0.17, 95%CI 0.12 to 0.23, 7 studies), and 18 months (RD 0.12, 95%CI 0.02 to 0.22, 3 studies). Of the nine studies included in the meta-analysis at the 6-month follow-up, four of them included participants with diabetes and depression. Of the seven studies included in the meta-analysis at the 12-month follow-up, three of them included participants with diabetes and depression. Of the three studies included in the meta-analysis at the 18-month follow-up, one included participants with diabetes and depression.

#### Cholesterol levels

One review reported on cholesterol levels. Smith et al. included two studies that reported on cholesterol levels. While a RCT found a reduction in low-density lipoprotein cholesterol, another RCT did not find any meaningful difference (SES ranges 0.22 to 0.26).

#### Diabetes clinical outcomes (HbA1c level)

Four reviews reported on clinical outcomes linked to diabetes. Atlantis et al., Huang et al., Smith et al., and Watson et al. reported on HbA1c levels and all of them found reduction of HbA1c levels favouring intervention.

Atlantis et al. found that collaborative care significantly reduced HbA1c levels in comparison to usual care (WMD -0.33, 95%CI -0.66 to 0.00%, 7 studies). Sensitivity analyses showed a slight decrease of pooled weighted mean difference (WMD) in the fixed effect model (WMD -0.21, 95%CI -0.37 to − 0.05, 7 studies). In addition, it was no longer statistically significant after the exclusion of each of the subgroups of studies (lower quality, outside of USA, integrated diabetes care, consideration of lifestyle risk factors, less than duration of 1 year).

Huang et al. calculated the mean difference (MD) and reported a non-significant reduction of HbA1c levels in favour of collaborative care (MD -0.13, 95%CI -0.46 to 0.19, 7 studies) at the end of follow-up. At 6-month follow-up, a non-significant reduction on HbA1c levels in favour of collaborative care (MD -0.06, 95%CI -0.24 to 0.12, 4 studies) was reported. At 12-month follow-up, a non-significant reduction on HbA1c levels in favour of collaborative care (MD -0.07, 95%CI -0.28 to 0.13, 4 studies) was reported.

Smith et al. reported that collaborative care significantly reduced HbA1c levels in comparison to usual care (WMD -0.33, 95%CI -0.66 to 0.00%, 7 studies).

Watson et al. found reduction in HbA1c levels in favour of collaborative care (MD 0.13, 95%CI -0.22 to 0.48, 3 studies) at 6 months. And at 12 months, reduction in HbA1c levels was still in favour of collaborative care (MD 0.24, 95%CI -0.14 to 0.62, 3 studies).

#### Effect of depression remission on HbA1c

One review reported on the effect of depression remission on diabetes. Atlantis et al. found that there was no association between the SMD in depression outcomes and the weighted mean difference (WMD) in HbA1c values. SMD for depression scores were unable to predict the WMD in HbA1c values (p − 0.828, coefficient 0.19, 95%CI -1.93 to 2.31, 7 studies).

#### Symptom improvement for depression and diabetes

One review reported on symptom improvement for depression and diabetes. Watson et al. reported greater depression symptom improvement scores in intervention groups at 6 months (MD 0.38, 95%CI 0.24 to 0.51, 5 studies), 12 months (MD 0.38, 95%CI 0.30 to 0.46, 5 studies), and 24 months (MD 0.18, 95%CI 0.10 to 0.26, 3 studies) compared to the control groups of usual care. Of the five studies included in the 6-month and 12-month meta-analyses, three of them included participants with diabetes and depression. Of the three studies included in the 24-month meta-analysis, two of them included participants with diabetes and depression. The benefits last through 24 months but a reduction in the magnitude of benefit was also mentioned.

#### Systolic blood pressure

One review reported on systolic blood pressure. Smith et al. reported on improvement of systolic blood pressure in favour of the intervention group (MD -3.10, 95%CI -7.26 to 1.06, 5 studies). The SESs varied from 0.01 to 1.12, but only one of the studies had an SES greater than 0.5.

### Process outcomes

#### Access and utilisation of healthcare services

Two reviews reported on access and utilisation of healthcare services. Smith et al. found five studies that reported on outcomes of health services utilisation, of which one RCT was of relevance to our review. The study did not find any difference in admission-related outcomes. However, it should be noted that the numbers of admission were very small.

Watson et al. reported that participants in the intervention group used more mental health services in comparison to the control group at 12 months (range: 42 to 84%; usual care range: 16 to 33%, 4 studies). Three of the studies included participants with depression and diabetes.

#### Costs

One review reported on costs linked to the intervention. Smith et al. provided data on five studies that reported on costs, of which one was of relevance to our review. An RCT found that the direct mean medical costs for TeamCare intervention for 12 months were $1224 USD per individual. In a later RCT by the same researchers, an economic analysis was conducted. They found that the intervention led to an increase of 114 days in depression-free days and an estimated difference of 0.335 quality-adjusted life years (QALYs) (95% CI -0.18 to 0.85).

#### Quality of care

One review reported on quality of care. Watson et al. reported on mental health treatment satisfaction in favour of collaborative care at 12 months (RD 0.205, 95%CI 0.112 to 0.299, 4 studies) and 24 months (RD 0.14, 95%CI 0.06 to 0.21, 3 studies). Of the four studies included in the 12-month meta-analysis, three of them included participants with diabetes and depression. Of the three studies included in the 24-month meta-analysis, one included participants with diabetes and depression.

## Integrated care for diabetes and hypertension

### Health outcomes

#### Achievement of control for hypertension

One review reported on control for hypertension. Joshi et al. reported on a before-after study and found that trained non-physician healthcare workers (NPHWs), without the input of physicians but assistance from treatment protocols, were able to achieve control of 68% of patients with hypertension and 82% of individuals with diabetes.

### Process outcomes

#### Access and utilisation of healthcare services

One review reported on access and utilisation of healthcare services. Joshi et al. found four studies that reported task-shifting improved access to healthcare at the community level. Of the four studies, three included participants with hypertension and diabetes. However, it must be noted that the metric to evaluate access was not described in most studies.

## Discussion

We conducted a scoping review on integration of care for hypertension and diabetes and identified five systematic reviews that were published from 2013 to 2016. Overall, collaborative care was better in comparison to usual care with regards to health and process outcomes for both depression and diabetes and hypertension and diabetes. Four of the included reviews focused on depression and diabetes. There was no significant difference in the outcomes for collaborative care for depression and diabetes with respect to risk to all-cause mortality and admission-related outcomes. Collaborative care has demonstrated better outcomes in depression scores, depression treatment response, depression remission, HbA1c levels, symptom improvements, systolic blood pressure, and mental health treatment satisfaction. There were mixed results with regard to cholesterol outcomes. No association was found between effect of depression remission and HbA1c levels. One included review focused on hypertension and diabetes. The use of task-shifting in collaborative care demonstrated improved access to care at the community level.

The majority of the primary studies included in the systematic reviews were conducted in high income countries, such as U.S.A., U.K., and Australia. Only one review [15] included primary studies conducted in low-and middle-income countries in SSA. None of the reviews focused on collaborative care for communicable and non-communicable diseases. Considering sub-Saharan Africa’s history of the HIV/AIDS and tuberculosis (TB) epidemics along with the recent rise of the quadruple burden of diseases (cardiovascular diseases, diabetes, chronic respiratory conditions, and cancer), it is important to direct future research on integration of care to cover comorbid communicable and non-communicable diseases. Possible outcomes to evaluate would include mortality rate, clinical outcomes specific to the individual diseases, symptom improvement, effect of one disease on another, quality of care, etc.

Our findings, particularly with respect to depression outcomes, were consistent to those reported in the current general literature on collaborative care programs for general, non-specific comorbid chronic disease programs. The evidence from primary studies and reviews demonstrated that collaborative care is more effective in improving short- and long-term depression outcomes and decreasing symptoms [30, 31]. Recent overviews noted that interventions targeted at specific combinations of comorbidities for patients with chronic illnesses were more likely to be more effective than interventions that target single specific diseases. In addition, these multi-component interventions were found to improve patient self-management outcomes and process-of-care behaviours [32, 33]. The agreement between our findings and that of other systematic reviews demonstrated that collaborative care, overall, are effective regardless of the comorbidities involved.

Integration of care has been demonstrated and described in multiple care models, such as the Chronic Care Model (CCM), collaborative care models, integrated/comprehensive-care programs, and other multi-component chronic-disease management. Although our scoping review had only included reviews that mainly focused on collaborative care, research on all relevant models demonstrating integration of care should be considered in relation to our findings. For example, a rapid synthesis of comparison of multi-component chronic-disease programs to disease-specific programs conducted by the McMaster Health Forum identified the following factors to be key facilitators to the implementation of CCM models: strong network support, increased communication between healthcare providers and organizations, creation of organizational culture that focuses on multidisciplinary and patient-centred care, recognition and commitment to efforts put forth by organizations and providers to induce change, implementation of structural and policy changes, leadership, and education for providers on CCM interventions and their effectiveness [33]. The CCM is defined as “an organizational approach to caring for people with chronic disease in a primary care setting.” It is a “population-based and creates practical, supportive, evidence-based interactions between an informed, activated patient and a prepared, proactive practice team [34].” It shares many similar elements with collaborative care, such as self-management support, delivery system design, decision support, and clinical information systems [34]. Noting the similar common components of both as well as the similarity of elements in the reviews used in our findings, the identified key facilitators to CCM may also be applicable to collaborative care model.

During the process of data extraction, we found that the interventions were unclear and not well-described, despite many of them being rather complex in nature. Furthermore, there was no consistency of descriptions of interventions between systematic reviews. Future systematic reviews on integrated care should describe their interventions in more detail.

In addition to the health and process outcomes that were identified in our review, Thota et al. also reviewed additional benefits such as the positive impact on patient’s job retention and work productivity, as well as their adherence to treatment [31]. To gain a more holistic review of the implications of collaborative care, this review also identified the potential harm brought on by the intervention and the potential barriers to implementation. By further supplementing the results of Thota et al.’s review with a systematic review by Watt et al., we can gain a better understand behind the causes of successes and failures behind integrated care. Watt et al. identified five themes on the facilitators and barriers to integration of HIV and chronic disease services and they included (1) ‘formal and informal productive relationships throughout the system’, (2) ‘need for adequate and appropriately skilled and incentivized health workers’, (3) ‘need for supportive institutional structures and dedicated resources’, (4) political leadership, ‘effective managerial oversight and organizational culture’, and (5) ‘placing the patient at the centre of service delivery’ [35]. From these studies, it could be seen that the potential implications of collaborative care should be considered in addition to its effectiveness in order to gain a comprehensive understanding of its feasibility. A future update of this review should also examine factors influencing implementation by including qualitative studies in addition to RCTs.

In assessing the overall risk of bias, ROBIS placed similar consideration for each of the domains as measured by the number/distribution of assessment items, while AMSTAR focused primarily on study selection, data collection, and synthesis and findings. With only an overlap of six assessment items which were included in both the AMSTAR and ROBIS tools, this suggested that the two tools included different aspects for consideration in their assessment. The higher number of discrepancies in assessment items for ROBIS may be attributed to its complex and highly divided grading scale. However, despite the high number of discrepancies, there was general agreement between the two reviewers on the overall risk of bias of the majority of systematic reviews.

There seemed to be no correlation between the PRISMA checklist score and the quality rating of a review. This reaffirmed the previous notion that the reporting checklist and the assessment tools are distinct instruments. The reporting checklist ensured the presence of relevant components of a systematic review but however, had no bearing on the quality of the components. Therefore, it is possible to achieve a high PRISMA score but a low quality in risk of bias score. Conversely, it is impossible to achieve a low PRISMA score but a high quality in risk of bias score, because the quality of the component cannot be assessed if it is not present.

The quality of the evidence and subsequently the results of a systematic review are unquestionably important, but it may be of little use to the reader if they do not apply to the reader’s question. Results of a study conducted under a certain setting may not necessarily be relevant to other settings. And as applicability of the results were not considered in either AMSTAR or ROBIS tools, additional instruments such as the SUPPORT tool should be used to supplement the risk of bias assessments [36].

With regards to the findings from the inter-rater reliability analysis, the poor inter-rater agreement suggested that assessing risk of bias using ROBIS as a first-time user may be quite difficult*.* This was consistent with the authors’ experiences with the tool. However, it was not surprising that the inter-rater agreement for ROBIS was much lower than that of AMSTAR given the finer discrimination the ROBIS rater must differentiate based on the data [37]. It should be noted that ROBIS adopted a non-linear, semi-ordinal/nominal scale in its rating answer options which could be justified in using either kappa or weighted kappa in our analysis. We decided to use kappa for both AMSTAR and ROBIS for the purpose of direct comparison. Given the poor inter-rater agreement, sensitivity analysis was considered but ultimately, we decided to not pursue it. As the decision to test for inter-rater reliability was included ad hoc, it was determined that it may be more suitable to keep the analysis simple and forego the sensitivity analysis.

## Robustness of our methodology

Similar to using ROBIS for risk of bias assessment on our included reviews, we also used its assessment criteria as a guide to ensure that our scoping review is conducted in a methodologically sound manner. For example, in phase 2 under the domain of study eligibility criteria, we ensured that we used appropriate and unambiguous predefined objectives and eligibility criteria. Next, under the domain for identification and selection of studies, we included a wide range of databases and additional search methods to identify all relevant reviews while selecting the studies in an independent and duplicate manner to minimize the risk of errors. In the subsequent domain of data collection and study appraisal, similar independent and duplicate efforts were made to minimize error in data collection and risk of bias assessment. And under the final domain of synthesis and findings, many of the criteria posed in the signalling questions did not apply as we did not conduct a meta-analysis. However, the synthesis included all relevant studies as it should.

## Limitations

This scoping review was limited by the small body of literature available on integration of care in delivery for hypertension and diabetes. Only five systematic reviews met the inclusion criteria set a priori. Therefore, this review should be considered as stimulation for further discussion and research on this matter. In addition, as previously mentioned, the majority of the included reviews were composed of primary studies held in higher income countries and thus the results may not be feasible or applicable to the CEBHA+ initiative which is aimed at a SSA context. With regards to the inter-rater reliability component of this paper, this analysis was determined ad hoc and the data collected was not optimized for this purpose. And further supporting/demonstrating the limitation of available literature, the *p* values were extremely underpowered with only five studies included in the analysis. It should be further noted that the raters had different levels of experience in using the risk of bias tools which may have resulted in a lower inter-rater agreement than *normal*.

## Conclusion

The findings from this scoping review have indicated a noticeable knowledge gap in integrated care in delivery-system interventions for co-morbid hypertension and diabetes present in the current literature. There is limited evidence from low-and middle-income countries. Future systematic reviews should assess the effect of integrated models of care for non-communicable diseases (such as diabetes and hypertension) and communicable diseases (such as HIV and TB). Systematic reviews should adequately report on the components of the interventions and assess clinical as well as system-level process outcomes such as access to health care, continuity of care, quality of care, cost of care, and user-views of care recipients.

From our comparison of AMSTAR and ROBIS tools, it could be seen that both were useful and more or less consistent in providing us with overall judgments about risk of bias. The purpose of such tools is to guide the risk of bias assessment and given that the most important thing is to simply consider risk of bias when considering evidence, both tools are adequate in achieving this objective.

## Additional files

Additional file 1: Search strategies. Search strategies for EMBASE, MEDLINE, Cochrane Library, Health Evidence. (DOCX 18 kb)

Additional file 2: ROBIS tool. Blank template of ROBIS tool. (DOCX 16 kb)

Additional file 3: AMSTAR tool. Blank template of AMSTAR tool. (DOCX 13 kb)

Additional file 4: PRISMA checklist. Blank template of PRISMA checklist tool. (DOCX 17 kb)

Additional file 5: Characteristics of excluded studies. List of excluded studies and reasons for exclusion. (DOCX 20 kb)

Additional file 6: Matrix table of studies included in depression reviews. Matrix table of primary studies included in depression reviews. (DOCX 13 kb)

Additional file 7: Matrix table of risk of bias items assessed in AMSTAR and ROBIS. Comparison of risk of bias items used in AMSTAR and ROBIS tools. (DOCX 13 kb)

Additional file 8: Inter-rater reliability from risk of bias assessment using ROBIS. Analysis of the degree of agreement between raters using the ROBIS tool. (DOCX 15 kb)

Additional file 9: Inter-rater reliability from risk of bias assessment using AMSTAR. Analysis of the degree of agreement between raters using the AMSTAR tool. (DOCX 13 kb)

## Abbreviations

AIDS: Acquired immune deficiency syndrome
AMSTAR: Assessing the methodological quality of systematic reviews tool
CCM: Chronic Care Model
CD: Communicable disease
CEBHA+: Collaboration for Evidence-Based Healthcare and Public Health in Africa
CI: Confidence interval
EMBASE: Excerpta Medica database
EMTREE: Excerpta Medica Tree
HIV: Human immunodeficiency virus
MD: Mean difference
MEDLINE: Medical Literature Analysis and Retrieval System Online
MeSH: Medical subject heading
NCD: Non-communicable disease
NPHW: Non-physician healthcare worker
PRISMA: Preferred Reporting Items for Systematic Reviews and Meta-Analyses
QALY: Quality-adjusted life year
RCT: Randomised controlled trial
RD: Risk difference
ROBIS: Risk of Bias of Systematic Reviews tool
RR: Response rate
SES: Standardised effect sizes
SMD: Standardised mean difference
SPSS: Statistical Package for the Social Sciences
SSA: Sub-Saharan Africa
TB: Tuberculosis
USD: United States Dollar
WMD: Weighted mean difference
YLD: Years lived with disability
