# Supplementary material for: Integration of care for hypertension and diabetes: a scoping review assessing the evidence from systematic reviews and evaluating reporting
Source: BMC Health Serv Res. 2018 Jun 20;18:481. doi: 10.1186/s12913-018-3290-8 (PMC6011271; doi:10.1186/s12913-018-3290-8)
Supplement: Supplementary file 1 — Search strategies. Search strategies for EMBASE, MEDLINE, Cochrane Library, Health Evidence. (DOCX 18 kb) [file 12913_2018_3290_MOESM1_ESM.docx]

**Additional file 1. Search strategies**

**Search strategy for EMBASE**

1. diabetes mellitus.mp. or exp diabetes mellitus/
2. diabet$,mp.
3. (non insulin* depend* or noninsulin* depend* or non insulin?depend* or noninsulin? depend*).mp. [mp=title, abstract, heading word, drug trade name, original title, device manufacturer, drug manufacturer, device trade name, keyword, floating subheading]
4. (insulin* depend* or insulin?depend*).mp. [mp=title, abstract, heading word, drug trade name, original title, device manufacturer, drug manufacturer, device trade name, keyword, floating subheading]
5. (IDDM or NIDDM or MODY or T1DM or T2DM or T1d or T2D).mp. [mp=title, abstract, heading word, drug trade name, original title, device manufacturer, drug manufacturer, device trade name, keyword, floating subheading]
6. 1 or 2 or 3 or 4 or 5
7. Exp hypertension/ or hypertension.mp.
8. Blood pressure.mp. or exp blood pressure/
9. 7 or 8
10. Comorbidity.mp. or exp comorbidity/
11. (comorbid$ or co-morbid$).mp. [mp=title, abstract, heading word, drug trade name, original title, device manufacturer, drug manufacturer, device trade name, keyword, floating subheading]
12. (multimorbid$ or multi-morbid$)mp. [mp=title, abstract, heading word, drug trade name, original title, device manufacturer, drug manufacturer, device trade name, keyword, floating subheading]
13. Chronic disease.mp. or exp chronic disease/
14. (chronic$ adj3 (disease? or ill$ or care or condition? or disorder? Or health$ or medication$ or syndrome$ or symptom$)).mp. [mp=title, abstract, heading word, drug trade name, original title, device manufacturer, drug manufacturer, device trade name, keyword, floating subheading]
15. ((coocur$ or co-occur$ or coexist$ or co-exist$ or multipl$) adj3 (disease? or ill$ or care or condition? or disorder$ or health$ or medication$ or symptom$ or syndrome$)).mp. [mp=title, abstract, heading word, drug trade name, original title, device manufacturer, drug manufacturer, device trade name, keyword, floating subheading]
16. 10 or 11 or 12 or 13 or 14 or 15
17. Integrated Health Care System.mp. or exp integrated health care system/
18. (integrat$ adj3 (care or service? or delivery or strategy$ or program$ or management)).mp. [mp=title, abstract, heading word, drug trade name, original title, device manufacturer, drug manufacturer, device trade name, keyword, floating subheading]
19. Health Care Delivery.mp. or exp health care delivery/
20. Integration.mp. or exp integration/
21. Patient Referral.mp. or exp patient referral/
22. Integrat$.mp.
23. (deliver$ adj3 (with or within or together)).mp. [mp=title, abstract, heading word, drug trade name, original title, device manufacturer, drug manufacturer, device trade name, keyword, floating subheading]
24. Bring together.mp.
25. Horizontal.mp. [mp=title, abstract, heading word, drug trade name, original title, device manufacturer, drug manufacturer, device trade name, keyword, floating subheading]
26. Vertical.mp. [mp=title, abstract, heading word, drug trade name, original title, device manufacturer, drug manufacturer, device trade name, keyword, floating subheading]
27. Coordinat$.mp. [mp=title, abstract, heading word, drug trade name, original title, device manufacturer, drug manufacturer, device trade name, keyword, floating subheading]
28. Co-ordinat$.mp. [mp=title, abstract, heading word, drug trade name, original title, device manufacturer, drug manufacturer, device trade name, keyword, floating subheading]
29. Link$.mp. [mp=title, abstract, heading word, drug trade name, original title, device manufacturer, drug manufacturer, device trade name, keyword, floating subheading] [mp=title, abstract, heading word, drug trade name, original title, device manufacturer, drug manufacturer, device trade name, keyword, floating subheading]
30. (multi$ adj team).mp. [mp=title, abstract, heading word, drug trade name, original title, device manufacturer, drug manufacturer, device trade name, keyword, floating subheading]
31. (multi$ adj2 (care or service or clinic)).mp. [mp=title, abstract, heading word, drug trade name, original title, device manufacturer, drug manufacturer, device trade name, keyword, floating subheading]
32. (multicare or multiservice or multiclinic).mp. [mp=title, abstract, heading word, drug trade name, original title, device manufacturer, drug manufacturer, device trade name, keyword, floating subheading]
33. Multiskill$.mp. [mp=title, abstract, heading word, drug trade name, original title, device manufacturer, drug manufacturer, device trade name, keyword, floating subheading]
34. Multi skill$.mp. [mp=title, abstract, heading word, drug trade name, original title, device manufacturer, drug manufacturer, device trade name, keyword, floating subheading]
35. Multitask$.mp. [mp=title, abstract, heading word, drug trade name, original title, device manufacturer, drug manufacturer, device trade name, keyword, floating subheading]
36. Multi task$.mp. [mp=title, abstract, heading word, drug trade name, original title, device manufacturer, drug manufacturer, device trade name, keyword, floating subheading]
37. 17 or 18 or 19 or 20 or 21 or 22 or 23 or 24 or 25 or 26 or 27 or 28 or 29 or 30 or 31 or 32 or 33 or 34 or 35 or 36
38. ((systematic adj2 (review* or overview* or synthesis or literature review* or evidence review*)) or methodology* review* or quantatitiv* review* or qualitative review* or overview or synthes* or metasynthes* or megasynthes*).mp. [mp=title, abstract, heading word, drug trade name, original title, device manufacturer, drug manufacturer, device trade name, keyword, floating subheading]
39. “review”/
40. Meta analysis/
41. Meta?analys$.mp.
42. (systematic$ adj5 review$).mp. [mp=title, abstract, heading word, drug trade name, original title, device manufacturer, drug manufacturer, device trade name, keyword, floating subheading]
43. (systematic$ adj5 overview$).mp. [mp=title, abstract, heading word, drug trade name, original title, device manufacturer, drug manufacturer, device trade name, keyword, floating subheading]
44. (quantiativ$ adj5 review$).mp. [mp=title, abstract, heading word, drug trade name, original title, device manufacturer, drug manufacturer, device trade name, keyword, floating subheading]
45. (quantitative$ adj5 overview$).mp. [mp=title, abstract, heading word, drug trade name, original title, device manufacturer, drug manufacturer, device trade name, keyword, floating subheading]
46. (methodologic$ adj5 review$).mp. [mp=title, abstract, heading word, drug trade name, original title, device manufacturer, drug manufacturer, device trade name, keyword, floating subheading]
47. (methodologic$ adj5 overview$).mp. [mp=title, abstract, heading word, drug trade name, original title, device manufacturer, drug manufacturer, device trade name, keyword, floating subheading]
48. (quantitative$ adj5 synthesi$).mp. [mp=title, abstract, heading word, drug trade name, original title, device manufacturer, drug manufacturer, device trade name, keyword, floating subheading]
49. 38 or 39 or 40 or 41 or 42 or 43 or 44 or 45 or 46 or 47 or 48
50. 6 or 9
51. 16 and 37 and 49 and 50

**Search strategy for MEDLINE**

1. diabetes mellitus.mp. or exp Diabetes Mellitus/
2. diabet$,mp.
3. (IDDM or NIDDM or MODY or T1DM or T2DM or T1d or T2D).mp. [mp=title, abstract, original title, name of substance word, subject heading word, keyword heading word, protocol supplementary concept word, rare disease supplementary concept word, unique identifier]
4. (non insulin$ depend* or noninsulin$ depend$ or non insulin?depend$ or noninsulin? depend$).mp. [mp=title, abstract, original title, name of substance word, subject heading word, keyword heading word, protocol supplementary concept word, rare disease supplementary concept word, unique identifier]
5. (insulin$ depend$ or insulin?depend$).mp. [mp=title, abstract, original title, name of substance word, subject heading word, keyword heading word, protocol supplementary concept word, rare disease supplementary concept word, unique identifier]
6. 1 or 2 or 3 or 4 or 5
7. exp Hypertension/ or hypertension.mp.
8. blood pressure.mp. or exp Blood Pressure/
9. ((elevat$ or high$ or rais$) adj3 (diastolic or systolic or arterial blood) adj pressure).mp. [mp=title, abstract, original title, name of substance word, subject heading word, keyword heading word, protocol supplementary concept word, rare disease supplementary concept word, unique identifier]
10. 7 or 8 or 9
11. comorbidity.mp. or exp Comorbidity/
12. (comorbid$ or co-morbid$).mp. [mp=title, abstract, original title, name of substance word, subject heading word, keyword heading word, protocol supplementary concept word, rare disease supplementary concept word, unique identifier]
13. (multimorbid$ or multi-morbid$).mp. [mp=title, abstract, original title, name of substance word, subject heading word, keyword heading word, protocol supplementary concept word, rare disease supplementary concept word, unique identifier]
14. (multidisease? or multi-disease? or (multiple adj (ill$ or disease? or condition? or syndrome$ or disorder?))).mp. [mp=title, abstract, original title, name of substance word, subject heading word, keyword heading word, protocol supplementary concept word, rare disease supplementary concept word, unique identifier]
15. chronic disease.mp. or exp Chronic Disease/
16. (chronic$ adj3 (disease? or ill$ or care or condition? or disorder$ or health$ or medication$ or syndrom$ or symptom$)).mp. [mp=title, abstract, original title, name of substance word, subject heading word, keyword heading word, protocol supplementary concept word, rare disease supplementary concept word, unique identifier]
17. ((coocur$ or co-occur$ or coexist$ or multipl$) adj3 (disease? or ill$ or care or condition? or disorder$ or health$ or medication$ or symptom$ or syndrome$)).mp. [mp=title, abstract, original title, name of substance word, subject heading word, keyword heading word, protocol supplementary concept word, rare disease supplementary concept word, unique identifier]
18. chronic$.mp.
19. 11 or 12 or 13 or 14 or 15 or 16 or 17 or 18
20. delivery of health care, integrated.mp. or exp “Delivery of Health Care, Integrated”/
21. comprehensive health care.mp. or exp Comprehensive Health Care
22. continuity of patient care.mp. or exp “Continuity of Patient Care”/
23. exp Patient-Centred Care/or patient-centred care.mp.
24. (integrat$ adj3 (care or service? or delivery or strategy$ or program$ or management)).mp. [mp=title, abstract, original title, name of substance word, subject heading word, keyword heading word, protocol supplementary concept word, rare disease supplementary concept word, unique identifier]
25. integrated programs.mp.
26. Delivery of Health Care.mp. or exp “Delivery of Health Care”/
27. (Referral and Consultation).mp. [mp=title, abstract, original title, name of substance word, subject heading word, keyword heading word, protocol supplementary concept word, rare disease supplementary concept word, unique identifier]
28. Interinstitutional Relations.mp. or exp Interinstitutional Relations/
29. Community-Institutional Relations.mp. or exp Community-Institutional Relations/
30. integrat$.mp.
31. (deliver$ adj3 (with or within or together)).mp. [mp=title, abstract, original title, name of substance word, subject heading word, keyword heading word, protocol supplementary concept word, rare disease supplementary concept word, unique identifier]
32. bring together.mp.
33. horizontal.mp.
34. vertical.mp. [mp=title, abstract, original title, name of substance word, subject heading word, keyword heading word, protocol supplementary concept word, rare disease supplementary concept word, unique identifier]
35. coordinat$.mp. [mp=title, abstract, original title, name of substance word, subject heading word, keyword heading word, protocol supplementary concept word, rare disease supplementary concept word, unique identifier]
36. co-ordinat$.mp. [mp=title, abstract, original title, name of substance word, subject heading word, keyword heading word, protocol supplementary concept word, rare disease supplementary concept word, unique identifier]
37. link$.mp. [mp=title, abstract, original title, name of substance word, subject heading word, keyword heading word, protocol supplementary concept word, rare disease supplementary concept word, unique identifier]
38. (multi$ adj team?).mp. [mp=title, abstract, original title, name of substance word, subject heading word, keyword heading word, protocol supplementary concept word, rare disease supplementary concept word, unique identifier]
39. (multi$ adj2 (care or service? or clinic?)).mp. [mp=title, abstract, original title, name of substance word, subject heading word, keyword heading word, protocol supplementary concept word, rare disease supplementary concept word, unique identifier]
40. (multicare or multiservice? or multiclinic?).mp. [mp=title, abstract, original title, name of substance word, subject heading word, keyword heading word, protocol supplementary concept word, rare disease supplementary concept word, unique identifier]
41. multiskill$.mp. [mp=title, abstract, original title, name of substance word, subject heading word, keyword heading word, protocol supplementary concept word, rare disease supplementary concept word, unique identifier]
42. multi skill$.mp. [mp=title, abstract, original title, name of substance word, subject heading word, keyword heading word, protocol supplementary concept word, rare disease supplementary concept word, unique identifier]
43. multitask$.mp. [mp=title, abstract, original title, name of substance word, subject heading word, keyword heading word, protocol supplementary concept word, rare disease supplementary concept word, unique identifier]
44. multi task$.mp. [mp=title, abstract, original title, name of substance word, subject heading word, keyword heading word, protocol supplementary concept word, rare disease supplementary concept word, unique identifier]
45. 20 or 21 or 22 or 23 or 24 or 25 or 26 or 27 or 28 or 29 or 30 or 31 or 32 or 33 or 34 or 35 or 36 or 37 or 38 or 39 or 40 or 41 or 42 or 43 or 44
46. ((systematic adj2 (review* or overview* or synthesis or literature review* or evidence review*)) or methodolg* review* or quantiativ* review* or qualitative review* or overview or synthes* or metasynthes* or megasynthes*).mp. [mp=title, abstract, original title, name of substance word, subject heading word, keyword heading word, protocol supplementary concept word, rare disease supplementary concept word, unique identifier]
47. (systematic review$ or methodology$ review$ or quantitative$ review$ or qualitativ$ review$ or overview$ or synthes$ or metasynthes$ or megasynthes$).mp. [mp=title, abstract, original title, name of substance word, subject heading word, keyword heading word, protocol supplementary concept word, rare disease supplementary concept word, unique identifier]
48. review literature.mp. or exp “Review”/
49. meta analysis.mp. or Meta-Analysis/
50. (meta-analys$ or meta analys$ or metaanlys$).mp. [mp=title, abstract, original title, name of substance word, subject heading word, keyword heading word, protocol supplementary concept word, rare disease supplementary concept word, unique identifier]
51. (systematic$ adj5 review$).mp. [mp=title, abstract, original title, name of substance word, subject heading word, keyword heading word, protocol supplementary concept word, rare disease supplementary concept word, unique identifier]
52. (systematic$ adj5 overview$).mp. [mp=title, abstract, original title, name of substance word, subject heading word, keyword heading word, protocol supplementary concept word, rare disease supplementary concept word, unique identifier]
53. (quantitativ$ adj5 review$).mp. [mp=title, abstract, original title, name of substance word, subject heading word, keyword heading word, protocol supplementary concept word, rare disease supplementary concept word, unique identifier]
54. (quantitativ$ adj5 overview$).mp. [mp=title, abstract, original title, name of substance word, subject heading word, keyword heading word, protocol supplementary concept word, rare disease supplementary concept word, unique identifier]
55. (quantitative$ adj5 synthesis$).mp. [mp=title, abstract, original title, name of substance word, subject heading word, keyword heading word, protocol supplementary concept word, rare disease supplementary concept word, unique identifier]
56. (methodologic$ adj5 review$).mp. [mp=title, abstract, original title, name of substance word, subject heading word, keyword heading word, protocol supplementary concept word, rare disease supplementary concept word, unique identifier]
57. (methodologic$ adj5 overview$).mp. [mp=title, abstract, original title, name of substance word, subject heading word, keyword heading word, protocol supplementary concept word, rare disease supplementary concept word, unique identifier]
58. 46 or 47 or 48 or 49 or 50 or 51 or 52 or 53 or 54 or 55 or 56 or 57
59. 6 or 10
60. 9 and 45 and 58 and 59

**Search strategy for Cochrane Library**

1. MeSH descriptor Diabetes mellitus explode all trees
2. diabet* in All Text
3. (IDDM in All Text or NIDDM in All Text or MODY in All Text or T1DM in All Text or T2DM in All Text or T1D in All Text or T2D in All Text)
4. ((non in All Text and insulin* in All Text and depend* in All Text) or (noninsulin* in All Text and depend* in All Text) or (non in All Text and insulin>depend* in All Text) or noninsulin?depend* in All Text)
5. ((insulin* in All Text and depend* in All Text) or insulin?depend* in All Text)
6. 1 or 2 or 3 or 4 or 5
7. MeSH descriptor Hypertension explode all trees
8. Hypertens* in Record Title
9. exp hypertension/
10. hypetens$.tw.
11. exp blood pressure/
12. ((elevat$ or high$ or rais$) adj3 (diastolic or systolic or arterial or blood) adj pressure).tw.
13. 7 or 8 or 9 or 10 or 11 or 12
14. MeSH descriptor Comorbidity explode all trees
15. (comorbid* or co-morbid* or multimorbid* or multi-morbid* or multidisease or multidiseases or multi-disease or multi-diseases):ti
16. MeSH descriptor Chronic Disease explode all trees
17. ((coocur* or co-occur* or coexist* or co-exist* or multipl*) near/2 (disease or diseases or ill* or care or condition or conditions or disorder* or health* or medication* or symptoms* or syndrome*)):ti,ab
18. 14 or 15 or 16 or 17
19. MeSH descriptor Delivery of Health Care, Integrated, this term only
20. MeSH descriptor Comprehensive Health Care, this term only
21. MeSH descriptor Continuity of Patient Care, this term only
22. MeSH descriptor Patient-Centred Care, this term only
23. (comprehensive NEXT health NEXT care):ti or (comprehensive NEXT health NEXT care):ab
24. (continuity NEAR/2 care):ti or (continuity NEAR/2 care):ab
25. (patient NEXT centred NEXT care):ti or (patient NEXT centered NEXT care):ab
26. (integrat* NEAR/3 (care or service* or delivery or staretg* or program* or management)):ti or (integrat* NEAR/3 (care or service* or delivery or strategy* or program* or management)):ab
27. MeSH descriptor Delivery of Health Care, this term only
28. MeSH descriptor Referral and Consultation, this term only
29. MeSH descriptor Interinstitutional Relations, this term only
30. (interact*):ti or (integrat*):ab
31. (bring NEXT together):ti or (bring NEXT together):ab
32. (horizontal or vertical):ti or (horizontal or vertical):ab
33. (coordinat*):ti or (coordinat*):ab
34. (co-ordinat*):ti or (co-ordinat*):ab
35. (link*):ti or (link*):ab
36. (multi* NEXT team*):ti or (multi* NEXT team*):ab
37. (multi* NEAR/2 (care or service* or clinic*)):ti or (multi* NEAR/2 (care or service* or clinic*)):ab
38. (multicare or multiservice* or multiclinic*):ti or (multicare or multiservice* or multiclinic*):ab
39. (multiskill* or multitask*):ti or (mltiskill* or multitask*):ab
40. (multi NEXT skill* or multi NEXT task*):ti or (multi NEXT skill* or multi NEXT task*):ab
41. 19 or 20 or 21 or 22 or 23 or 24 or 25 or 26 or 27 or 28 or 29 or 30 or 31 or 32 or 33 or 34 or 35 or 36 or 37 or 38 or 39 or 40
42. 6 or 13 and 18 and 41

**Search strategy for Health Evidence**

[diabetes OR hypertension AND comorbidity AND (integration of care)] AND limit: Review Type = Meta-analysis, Systematic review of reviews
