# Supplementary material for: Integration of care for hypertension and diabetes: a scoping review assessing the evidence from systematic reviews and evaluating reporting
Source: BMC Health Serv Res. 2018 Jun 20;18:481. doi: 10.1186/s12913-018-3290-8 (PMC6011271; doi:10.1186/s12913-018-3290-8)
Supplement: Supplementary file 3 — AMSTAR tool. Blank template of AMSTAR tool. (DOCX 13 kb) [file 12913_2018_3290_MOESM3_ESM.docx]

**Additional file 3. AMSTAR tool**

| **Item** | **Yes/No/Can’t answer/Is not applicable** |
| --- | --- |
| 1. Was an ‘a priori’ design provided? |  |
| 1. Was there duplicate study selection and data extraction? |  |
| 1. Was a comprehensive literature search performed? |  |
| 1. Was the status of publication (i.e. grey literature) used as an inclusion criterion? |  |
| 1. Was a list of studies (included and excluded) provided? |  |
| 1. Were the characteristics of the included studies provided? |  |
| 1. Was the scientific quality of the included studies assessed and documented? |  |
| 1. Was the scientific quality of the included studies used appropriately in formulating conclusions? |  |
| 1. Were the methods used to combine the findings of studies appropriate? |  |
| 1. Was the likelihood of publication bias assessed? |  |
| 1. Was conflict of interest included? |  |

|  |
| --- |
|  |
|  |
