# Supplementary material for: Integration of care for hypertension and diabetes: a scoping review assessing the evidence from systematic reviews and evaluating reporting
Source: BMC Health Serv Res. 2018 Jun 20;18:481. doi: 10.1186/s12913-018-3290-8 (PMC6011271; doi:10.1186/s12913-018-3290-8)
Supplement: Supplementary file 5 — Characteristics of excluded studies. List of excluded studies and reasons for exclusion. (DOCX 20 kb) [file 12913_2018_3290_MOESM5_ESM.docx]

**Additional file 5. Characteristics of excluded studies**

| **Study** | **Reason for exclusion** |
| --- | --- |
| Barlow 2007,^1^ Bazian 2005,^2^ Bleich 2011,^3^ Cabana 2004,^4^ Coster 2009,^5^ Dashora 2011,^6^ Durao 2015,^7^ Greer 2016,^8^ Grover 2015,^9^ Health Quality Ontario 2012,^10^ Health Quality Ontario 2013,^11^ Hill 2010,^12^ Kirsh 2008,^13^ Kripalani 2007,^14^ Lall 2014,^15^ Loney-Hutchinson 2009,^16^ Martinez-Gonzalez 2014,^17^ Pare 2010,^18^ Rahimi Naini 2014,^19^ Roberts 2012,^20^ Rossom 2016,^21^ Sampsel 2007,^22^ Singh 2005,^23^ Steigerwalt 2008,^24^ Team 2011,^25^ Wang 2014,^26^ Warsi 2004,^27^ Yasmin 2016,^28^ Zimbudzi 2015 ^29^ | Did not meet our systematic review criteria |
| Crowley 2011,^30^ Desveaux 2014,^31^ Flodgren 2015,^32^ Gallagher 2010,^33^ Hersh 2011,^34^ Jang 2012,^35^ Kuipers 2013,^36^ Li 2013,^37^ Matire 2010,^38^ Matire 2005,^39^ McDermott 2013,^40^ McMillan 2013,^41^ Pearson 2003,^42^ Polisena 2009,^43^ Portz 2016,^44^ Quinones 2014,^45^ Rachas 2016,^46^ Rees 2009,^47^ Rosser 2009,^48^ Ru 2010,^49^ Ruby 2015,^50^ Scheuner 2008,^51^ Siantz 2014,^52^ Small 2013,^53^ Smith 2008,^54^ Smith 2007,^55^ Song 2015,^56^ Stellefson 2013,^57^ Tennant 2007,^58^ Vaes 2013,^59^ van den Brink 2013,^60^ van Hecke 2016,^61^ van Wijk 2005 ^62^ | Intervention not relevant |
| Jensen ^63^ | Full text unavailable |

^1^ Barlow J, Singh D, Bayer S, Curry R. A systematic review of the benefits of home telecare for frail elderly people and those with long-term conditions. J Telemed Telecare. 2007;13:172-9.

2 Bazian Ltd. The effects of education on patient adherence to medication. Evidence-Based Healthcare & Public Health. 2005;9:398-404.

3 Bleich SN, Koehlmoos TLP, Rashid M, Peters DH, Anderson G. Noncommunicable chronic disease in Banglasdesh: Overview of existing programs and priorities going forward. Health Policy. 2011;100(2-3):282-9.

4 Cabana MD, Jee SH. Does continuity of care improve patient outcomes? J Fam Pract. 2004;53(12):974-80.

5 Coster S, Norman I. Cochrane reviews of educational and self-management interventions to guide nursing practice: A review. Int J Nurs Stud. 2009;46:508-28.

6 Dashora U, Radia K, Radia C. Integrated care: Improving glycaemic control in joint clinics. Prim Care Diabetes. 2011;13(6):369-74.

7 Durao S, Ajumobi O, Kredo T, et al. Evidence insufficient to confirm the value of population screening for diabetes and hypertension in low- and middle-income settings. SAMJ. 2015;105(2):98-102.

8 Greer N, Bolduc J, Geurkink E, et al. Pharmacist-led chronic disease management: A systematic review of effectiveness and harms compared with usual care. Ann Intern Med. 2016;165:30-40.

9 Grover A, Joshi A. An overview of chronic disease models: A systematic literature review. 2015;7(2):210-27.

10 Health Quality Ontario. Specialized community-based care: An evidence-based analysis. Ont Health Technol Assess Ser. 2012;12(20):1-60.

11 Health Quality Ontario. Specialized nursing practice for chronic disease management in the primary care setting: An evidence-based analysis. Ont Health Technol Assess Ser. 2013;13(10):1-66.

12 Hill RD, Luptak MK, Rupper RW, et al. Review of veterans health administration telemedicine interventions. Am J Manag Care. 2010;16(12 Spec No.):e302-10.

13 Kirsch SR, Aron DC. Integrating the chronic-care model and the ACGME competencies: Using shared medical appointments to focus on systems-based practice. Qual Saf Health Care. 2008;17:15-9.

^14^ Kripalani S, Yao X, Haynes B. Interventions to enhance medication adherence in chronic medical conditions: A systematic review. Arch Intern Med. 2007;167:540-50.

15 Lall D, Prabhakaran D. Organization of primary health care for diabetes and hypertension in high, low and middle income countries. Expert Rev Cardiovasc Ther. 2014;12(8):987-95.

16 Loney-Hutchinson LM, Provilus AD, Jean-Louis G, Zizi F, Ogedegbe O, McFarlane SI. Group visits in the management of diabetes and hypertension: Effect on glycemic and blood pressure control. Curr Diab Rep. 2009;9:238-42.

17 Martinez-Gonzalez NA, Berchtold P, Ullman K, Busato A, Egger M. Integrated care programmes for adults with chronic conditions: A meta-review. Int J Qual Health Care. 2014;26(5):561-70.

18 Pare G, Moqadem K, Pineau G, St-Hillaire C. Clinical effects of home telemonitoring in the context of diabetes, asthma, heart failure and hypertension: A systematic review. J Med Internet Res. 2010;12(2):e21-35.

19 Rahimi Naini S, Fuchs M. Non-alcoholic fatty liver disease in patients with diabetes mellitus. Expert Rev Endocrinol Metab. 2014;9(5):503-14.

20 Roberts RG, Gask L, Arndt B, et al. Depression and diabetes: The role and impact of models of health care systems. J Affect Disord. 2012;142S1:S80-8.

21 Rossom RC, Solberg LI, Magnan S, et al. Impact of a national collaborative care initiative for patients with depression and diabetes or cardiovascular disease. Gen Hosp Psychiatry. 2016. http://dx.doi.org/10.1016/j.genhosppsych.2016.05.006.

22 Sampsel S, May J. Assessment and management of obesity and comorbid conditions. Dis Manag. 2007;10(5):252-65.

23 Singh D, Surrey and Sussex Primary Care Trust Alliance. Transforming chronic care: A systematic review of the evidence. Evid Based Cardiovasc Med. 2005;9:91-4.

24 Steigerwalt S. Management of hypertension in diabetic patients with chronic kidney disease. Diabetes Spectr. 2008;21(1):30-6.

2^5^ Team V, Canaway R, Manderson L. Integration of complementary and alternative medicine information and advice in chronic disease management guidelines. Aust J Prim Health. 2011;17:142-9.

26 Wang J, Wang Y, Wei C, et al. Smartphone interventions for long-term health management of chronic diseases: An integrative review. Telemed J E Health. 2014;20(6):570-83.

27 Warsi A, Wang PS, LaValley MP, Avorn J, Solomon DH. Self-management education programs in chronic disease. Arch Intern Med. 2004;164:1641-9.

2^8^ Yasmin F, Banu B, Zakir SM, Sauerborn R, Ali L, Souares A. Positive influence of short message service and voice call interventions on adherence and health outcomes in case of chronic disease care: A systematic review. BMC Med Inform Decis Mak. 2016;16:46-59.

^29^ Zimbudzi E, Lo C, Misso M, Ranasinha S, Zoungas S. Effectiveness of management models for facilitating self-management and patient outcomes in adults with diabetes and chronic kidney disease. Syst Rev. 2015;4:81-8.

^30^ Crowley R, Wolfe I, Lock K, McKee M. Improving the transition between paediatric and adult healthcare: A systematic review. Arch Dis Child. 2011;96:548-53.

31 Desveaux L, Beauchamp M, Goldstein R, Brooks D. Community-based exercise programs as a strategy to optimize function in chronic disease. Med Care. 2014;52:216-26.

32 Flodgren G, Rachas A, Farmer AJ, Inzitari M, Shepperd S. Interactive telemedicine: Effects on professional practice and health care outcomes (Review). Cochrane Database Syst Rev. 2015;9. DOI: 10.1002/14651858.CD002098.pub2.

33 Gallagher H, de Lusignan S, Harris K, Cates C. Quality-improvement strategies for the management of hypertension in chronic kidney disease in primary care: A systematic review. Br J Gen Pract. 2010:e258-65.

34 Hersh WR, Helfand M, Wallace J, et al. Clinical outcomes resulting from telemedicine interventions: A systematic review. BMC Med Inform Decis Mak. 2001;1:5-12.

35 Jang Y, Yoo H. Self-management programs based on the social cognitive theory for Koreans with chronic disease: A systematic review. Contemp Nurse. 2012;40(2):147-59.

36 Kuijpers W, Groen WG, Aaronson NK, van Harten WH. A systematic review of web-based interventions for patient empowerment and physical activity in chronic diseases: Relevance for cancer survivors. J Med Internet Res. 2013;15(2):e37-55.

37 Li W, Guo H, Li H, Wang L, Fu H, Wang X. Integration of traditional Chinese medicines and western medicines for treating diabetes mellitus with coronary heart disease: A systematic review. J Altern Complement Med. 2013;19(6):492-500.

38 Martire LM, Schulz R, Helgeson VS, Small BJ, Saghafi EM. Review and meta-analysis of couple-oriented interventions for chronic illness. Ann Behav Med. 2010;40:325-42.

39 Martire LM. The “relative” efficacy of involving family in psychosocial interventions for chronic illness: Are there added benefits to patients and family members? Database of Abstracts of Reviews of Effects. 2005. Available at: https://www.ncbi.nlm.nih.gov/pubmedhealth/PMH0022638/?report=printable

40 McDermott MS, While AE. Maximizing the healthcare environment: A systematic review exploring the potential of computer technology to promote self-management of chronic illness in healthcare settings. Patient Educ Couns. 2013;92:13-22.

41 McMillan SS, Kendall E, Sav A, et al. Patient-centered approaches to health care: A systematic review of randomized controlled trials. 2013;70(6):567-96.

42 Pearson S, Ross-Degnan D, Payson A, Soumerai SB. Changing medication use in managed care: A critical review of the available evidence. Am J Manag Care. 2003;9(11):715-31.

43 Polisena J, Coyle D, Coyle K, McGill S. Home telehealth for chronic disease management: A systematic review and an analysis of economic evaluations. Int J Technol Assess Health Care. 2009;25(3):339-49.

^44^ Portz JD, Miller A, Foster B, Laudeman L. Persuasive features in health information technology interventions for older adults with chronic diseases: A systematic review. Health Technol. 2016;6:89-99.

45 Quinones AR, Richardson J, Freeman M, et al. Educational group visits for the management of chronic health conditions: A systematic review. Patients Educ Couns. 2014;95:3-29.

46 Rachas A, Lefeuvre D, Meyer L, et al. Evaluating continuity during transfer to adult care: A systematic review. Pediatrics. 2016;138(1):e20160256.

47 Rees S, Williams A. Promoting and supporting self-care management for adults living in the community with physical chronic illness: A systematic review of the effectiveness and meaningfulness of the patient-practitioner encounter. JBI Database System Rev Implement Rep. 2009;7(13):492-582.

48 Rosser BA, Vowles KE, Keogh E, Eccleston C, Mountain GA. Technologically-assisted behavior change: A systematic review of studies of novel techonologies for the management of chronic illness. J Telemed Telecare. 2009;15:327-38.

49 Ru TZ, Hong EPW, Hegney DG. A qualitative systematic review on the experiences of self-management in community-dwelling older women living with chronic illness. JBI Libr Syst Rev. 2011;9(62):2778-828.

50 Ruby A, Knight A, Perel P, Blanchet K, Roberts B. The effectiveness of interventions for non-communicable diseases in humanitarian crises: A systematic review. PLoS ONE. 2015;10(9):e0138303. DOI:10.1371/journal.pone.0138303.

51 Scheuner MT, Sieverding P, Shekelle PG. Delivery of genomic medicine for common chronic adult diseases. JAMA. 2008;299(11):1320-34.

52 Siantz E, Aranda M. Chronic disease self-management interventions for adults with serious mental illness: A systematic review of the literature. Gen Hosp Psychiatry. 2014;36:233-44.

53 Small N, Blickem C, Blakeman T, Panagioti M, Chew-Graham CA, Bower P. Telephone based self-management support by ‘lay health workers’ and ‘peer support workers’ to prevent and manage vascular diseases: A systematic review and meta-analysis. BMC Health Serv Res. 2013;13:533-49.

54 Smith SM, Allwright S, O’Dowd T. Does sharing care across the primary-specialty interface improve outcomes in chronic disease? A systematic review. Am J Manag Care. 2008;14(4):213-24.

55 Smith SM, Allwright S, O’Dowd T. Effectiveness of shared care across the interface between primary and specialty care in chronic disease management (Review). 2007;3. DOI: 10.1002/14651858.CD004910.pub2.

56 Song HJ, Choi SM, Seo H, Lee H, Son H, Lee S. Self-administered foot reflexology for the management of chronic health conditions: A systematic review. J Altern Complement Med. 2015;21(2):69-75.

57 Stellefson M, Chaney B, Barry AE, et al. Web 2.0 chronic disease self-management for older adults: A systematic review. J Med Internet Res. 2013;15(2):e35-48.

58 Tennant R, Mohammed MA, Coleman JJ, Martin U. Monitoring patients using control charts: A systematic review. Int J Qual Health Care. 2007;19(4):187-94.

59 Vaes AW, Cheung A Atakhorrami M, et al. Effect of ‘activity monitor-based’ counseling on physical activity and health-related outcomes in patients with chronic diseases: A systematic review and meta-analysis. Ann Med. 2013;45:397-412.

60 van den Brink AMA, Gerritsen DL, Oude Voshaar RC, Koopmans RTCM. Residents with mental-physical multimorbidity living in long-term care facilities: Prevalence and characteristics. A systematic review. 2013;25(4):531-48.

61 van Hecke A, Heinen M, Fernandez-Ortega P, et al. Systematic literature review on effectiveness of self-management support interventions in patients with chronic conditions and low socio-economic status. J Adv Nurs. 2016. DOI:10.1111/jan.13159.

62 van Wijk BLG, Klungel OH, Heerdink ER, de Boer A. Effectiveness of interventions by community pharmacists to improve patient adherence to chronic medication: A systematic review. Ann Pharmacother. 2005;39:319-28.

^63^ Jensen A.E., Skursky N., Beyrouty M., Bennett K., Schwartz M.D., Sherman S. Panel management in primary care: A systematic review. Journal of General Internal Medicine 2014 April 2014;29:S163^.^
