# Supplementary material for: Integration of care for hypertension and diabetes: a scoping review assessing the evidence from systematic reviews and evaluating reporting
Source: BMC Health Serv Res. 2018 Jun 20;18:481. doi: 10.1186/s12913-018-3290-8 (PMC6011271; doi:10.1186/s12913-018-3290-8)
Supplement: Supplementary file 6 — Matrix table of studies included in depression reviews. Matrix table of primary studies included in depression reviews. (DOCX 13 kb) [file 12913_2018_3290_MOESM6_ESM.docx]

**Additional file 6. Matrix table of studies included in depression reviews**

| **Our Included SRs →** | Atlantis 2014 [21] | Huang 2013 [22] | Smith 2016 [24] | Watson 2013 [25] |
| --- | --- | --- | --- | --- |
| **Studies in SR ↓** |  |  |  |  |
| Bogner 2008 |  |  | X |  |
| Bogner 2010 | X | X |  |  |
| Bogner 2012 | X | X |  |  |
| Ciechanowski 2006 |  | X |  | X |
| Coventry 2015 |  |  | X |  |
| Ell 2010 | X |  |  | X |
| Ell 2011 |  | X |  | X |
| Hay 2012 |  |  |  | X |
| Katon 2004 | X | X |  | X |
| Katon 2006 |  |  |  | X |
| Katon 2008 |  |  |  | X |
| Katon 2010 | X | X | X | X |
| Kinder 2006 |  | X |  | X |
| Lin 2006 |  |  |  | X |
| Lynch 2014 |  |  | X |  |
| Morgan 2013 | X |  | X |  |
| Simon 2007 |  |  |  | X |
| Wakefield 2012 |  |  | X |  |
| Williams 2004 | X | X |  | X |
