# Supplementary material for: Integration of care for hypertension and diabetes: a scoping review assessing the evidence from systematic reviews and evaluating reporting
Source: BMC Health Serv Res. 2018 Jun 20;18:481. doi: 10.1186/s12913-018-3290-8 (PMC6011271; doi:10.1186/s12913-018-3290-8)
Supplement: Supplementary file 7 — Matrix table of risk of bias items assessed in AMSTAR and ROBIS. Comparison of risk of bias items used in AMSTAR and ROBIS tools. (DOCX 13 kb) [file 12913_2018_3290_MOESM7_ESM.docx]

**Additional file 7. Matrix table of risk of bias items assessed in AMSTAR and ROBIS**

| **Risk of bias assessment item** | **AMSTAR** | **ROBIS** |
| --- | --- | --- |
| STUDY ELIGIBILTY |  |  |
| Pre-defined objectives and eligibility criteria/ ‘a priori’ design | X | X |
| Appropriateness of eligibility criteria |  | X |
| Ambiguity of eligibility criteria |  | X |
| Restriction of eligibility criteria |  | X |
| Appropriateness of restrictions of eligibility criteria |  | X |
| STUDY SELECTION |  |  |
| Duplicate study selection and data extraction | X |  |
| Appropriate range of databases for search | X | X |
| Status of publication used as inclusion criterion | X |  |
| Additional search methods |  | X |
| Terms and structure of search strategy |  | X |
| Appropriateness of search restrictions |  | X |
| Minimisation of error in selection |  | X |
| List of inclusion and exclusion studies | X |  |
| DATA COLLECTION |  |  |
| Minimisation of error in data collection |  | X |
| Availability of sufficient study characteristics | X | X |
| Collection of relevant study results for synthesis |  | X |
| Formal assessment of risk of bias | X | X |
| Minimisation of error in risk of bias assessment |  | X |
| SYNTHESIS AND FINDINGS |  |  |
| Inclusion of all appropriate studies |  | X |
| Explanation of all pre-defined analyses |  | X |
| Appropriateness of synthesis | X | X |
| Addressing of heterogeneity |  | X |
| Robustness of findings |  | X |
| Addressing of biases in primary studies | X | X |
| Scientific quality of included studies used in formulation of conclusions | X |  |
| OTHER |  |  |
| Conflict of interest | X |  |
