# Supplementary material for: Integration of care for hypertension and diabetes: a scoping review assessing the evidence from systematic reviews and evaluating reporting
Source: BMC Health Serv Res. 2018 Jun 20;18:481. doi: 10.1186/s12913-018-3290-8 (PMC6011271; doi:10.1186/s12913-018-3290-8)
Supplement: Supplementary file 8 — Inter-rater reliability from risk of bias assessment using ROBIS. Analysis of the degree of agreement between raters using the ROBIS tool. (DOCX 15 kb) [file 12913_2018_3290_MOESM8_ESM.docx]

**Additional file 8. Inter-rater Reliability from Risk of Bias Assessment Using ROBIS**

| **Domain** | **Question** | **Kappa Value** | **Standard Error** | **95% CI** | **p Value** | **Percent Agreement** |
| --- | --- | --- | --- | --- | --- | --- |
| 1 | 1 | -0.111 | 0.177 | -0.458 to 0.235 | 0.661 | 0.2 |
|  | 2 | 0.231 | 0.141 | -0.046 to 0.508 | 0.171 | 0.6 |
|  | 3 | 0.063 | 0.314 | -0.554 to 0.679 | 0.836 | 0.4 |
|  | 4 | 0.167 | 0.124 | -0.077 to 0.410 | 0.025 | 0.2 |
|  | 5 | 0.500 | 0.229 | 0.051 to 0.949 | 0.012 | 0.4 |
| 2 | 1 | 0.444 | 0.291 | -0.127 to 1.000 | 0.079 | 0.6 |
|  | 2 | -0.111 | 0.263 | -0.627 to 0.405 | 0.626 | 0.2 |
|  | 3 | 0.286 | 0.334 | -0.369 to 0.940 | 0.361 | 0.6 |
|  | 4 | 0.091 | 0.085 | -0.075 to 0.257 | 0.361 | 0.2 |
|  | 5 | 0 | 0 | 0 | 1.000 | 0.2 |
| 3 | 1 | 0.048 | 0.056 | -0.063 to 0.158 | 0.576 | 0.2 |
|  | 2 | -0.250 | 0.186 | -0.615 to 0.115 | 0.402 | 0.4 |
|  | 3 | 0 | 0.071 | -0.139 to 0.139 | 1.000 | 0.2 |
|  | 4 | 0.167 | 0.124 | -0.077 to 0.410 | 0.402 | 0.4 |
|  | 5 | 0.444 | 0.055 | 0.336 to 0.553 | 0.025 | 0.8 |
| 4 | 1 | -0.053 | 0.142 | -0.331 to 0.225 | 0.709 | 0.2 |
|  | 2 | 0 | 0.122 | -0.240 to 0.240 | 1.000 | 0.2 |
|  | 3 | -0.429 | 0.379 | -1.000 to 0.314 | 0.171 | 0.2 |
|  | 4 | -0.154 | 0.097 | -0.344 to 0.037 | 0.361 | 0.4 |
|  | 5 | -0.087 | 0.079 | -0.242 to 0.068 | 0.361 | 0.0 |
|  | 6 | 0 | 0.187 | -0.367 to 0.367 | 1.000 | 0.2 |
|  | A | 0.412 | 0.246 | -0.071 to 0.895 | 0.125 | 0.6 |
|  | B | Cannot be computed | Cannot be computed | Cannot be computed | Cannot be computed | 0.6 |
|  | C | -0.111 | 0.216 | -0.534 to 0.311 | 0.661 | 0.2 |
