# Supplementary material for: Integration of care for hypertension and diabetes: a scoping review assessing the evidence from systematic reviews and evaluating reporting
Source: BMC Health Serv Res. 2018 Jun 20;18:481. doi: 10.1186/s12913-018-3290-8 (PMC6011271; doi:10.1186/s12913-018-3290-8)
Supplement: Supplementary file 9 — Inter-rater reliability from risk of bias assessment using AMSTAR. Analysis of the degree of agreement between raters using the AMSTAR tool. (DOCX 13 kb) [file 12913_2018_3290_MOESM9_ESM.docx]

**Additional file 9. Inter-rater Reliability from Risk of Bias Assessment Using AMSTAR**

| **Question** | **Kappa Value/Weighted** | **Standard Error** | **95% CI** | **p Value** | **Percent Agreement** |
| --- | --- | --- | --- | --- | --- |
| 1 | 0.375 | 0.086 | 0.207 to 0.543 | 0.025 | 0.6 |
| 2 | 0.231 | 0.141 | -0.046 to 0.508 | 0.171 | 0.6 |
| 3 | Cannot be computed | Cannot be computed | Cannot be computed | Cannot be computed | 1.0 |
| 4 | 0.286 | 0.183 | -0.072 to 0.643 | 0.073 | 0.4 |
| 5 | 1.000 | 0.000 | 1.000 to 1.000 | 0.025 | 1.0 |
| 6 | Cannot be computed | Cannot be computed | Cannot be computed | Cannot be computed | 1.0 |
| 7 | 0.000 | Cannot be computed | Cannot be computed | 1.000 | 0.8 |
| 8 | 0.286 | 0.274 | -0.251 to 0.822 | 0.361 | 0.6 |
| 9 | Cannot be computed | Cannot be computed | Cannot be computed | Cannot be computed | 1.0 |
| 10 | 0.545 | 0.362 | -0.164 to 1.000 | 0.171 | 0.8 |
| 11 | -0.364 | 0.272 | -0.896 to 0.169 | 0.361 | 0.4 |
